# Usefulness and limitations of sample pooling for environmental DNA metabarcoding of freshwater fish communities

**DOI:** 10.1038/s41598-017-14978-6

**Published:** 2017-11-01

**Authors:** Hirotoshi Sato, Yuki Sogo, Hideyuki Doi, Hiroki Yamanaka

**Affiliations:** 1 Department of Environmental Solution Technology, Facility of Science & Technology, Ryukoku University, Seta-Oe, Otsu, 520-2194 Shiga, Japan; 2 Graduate School of Simulation Studies, University of Hyogo, Minatojima-minamimachi, Kobe, 650-0047 Japan

## Abstract

Environmental DNA (eDNA) metabarcoding has been used increasingly to assess biodiversity of aquatic vertebrates. However, there still remains to be developed a sampling design of eDNA metabarcoding that can ensure high detection rates of species with minimum total survey effort, especially for large-scale surveys of aquatic organisms. We here tested whether pooling of eDNA samples can be used to evaluate biodiversity of freshwater fishes in four satellite lakes of Lake Biwa, Japan. Fish communities detected by eDNA metabarcoding of the mitochondrial 12S region were compared between the individual and pooled samples. In the individual samples, 31, 22, 33, and 31 fish lineages (proxies for species) were observed at the respective sites, within which moderate spatial autocorrelation existed. In the pooled samples, 30, 20, 29, and 27, lineages were detected, respectively, even after 15 PCR replicates. Lineages accounting for < 0.05% of the total read count of each site’s individual samples were mostly undetectable in the pooled samples. Moreover, fish communities detected were similar among PCR replicates in the pooled samples. Because of the decreased detection rates, the pooling strategy is unsuitable for estimating fish species richness. However, this procedure is useful potentially for among-site comparison of representative fish communities.

## Introduction

Knowledge of species distribution is essential for understanding community dynamics and biodiversity patterns, and for planning management and conservation of threatened and endangered species^1–3^. However, a precise estimation of species distribution is often difficult and inefficient, particularly in aquatic systems where most organisms are not visible owing to complex habitat topology and vegetation. Moreover, field monitoring sometimes appears to be destructive to the target species or ecosystem under study^4^. To overcome such limitations, there has been a need for alternative approaches.

Recently, environmental DNA (eDNA) techniques have been developed for species detection of aquatic macroorganisms^4–8^. In contrast to conventional survey methods, eDNA offers the advantages of being noninvasive and potentially more sensitive at low population densities of target organisms^9–11^. In particular, aquatic environments are suitable for applying eDNA techniques, where eDNA is distributed more homogeneously in water than in soil or other sediments^12^. To date, the analysis of eDNA using gel electrophoresis or quantitative PCR (qPCR) has proven highly successful for targeted detection of one or a few species inhabiting various aquatic environments^10,11,13–17^. Although these PCR-based approaches are powerful tools for monitoring target species, they cannot be used for assessing community composition of organisms.

An alternative approach is eDNA metabarcoding, which involves parallel sequencing of whole communities of organisms, and thereby offers comprehensive and efficient tools for assessing total biodiversity and community composition^8,18,19^. Although several technical and methodological challenges remain (e.g., primer biases, sequencing artifacts, contamination, misidentification of species, and sampling biases), eDNA metabarcoding has great advantages in terms of speed, cost per sample, coverage, and independence of taxonomic expertise compared to conventional morphology-based surveys^8,18,20–22^.

An increasing number of studies have performed eDNA metabarcoding for species detection, biodiversity assessment, and relative quantification of aquatic macroorganisms^8,18,19,21^. These studies first focused on describing fish communities in tanks or aquaria, and thereby they confirmed that the method is highly sensitive for detecting rare species^21,23–25^. More recently, metabarcoding has been used successfully to assess biodiversity and community structure of various macroorganisms in natural settings, including freshwater fishes^26–29^, marine fishes^30–33^, amphibians^34^, mammals^31,35^, and freshwater invertebrates^36^. Moreover, eDNA metabarcoding approaches potentially can be extended to explore large-scale spatiotemporal variations of community structure in aquatic ecosystems, unravelling trends linked to environmental variables or to human impacts^8,37^.

To assess biodiversity and community composition of aquatic vertebrates using eDNA metabarcoding, the sampling design not only must be effective but also efficient, especially for a large-scale study of communities over space and time. Use of small sample sizes may lead to underestimation of biodiversity because recent eDNA metabarcoding studies indicated that eDNA in aquatic systems appears to be spatially autocorrelated at large spatial scales^31,33,36^ (but also see^38^). Another labor-saving method is pooling of samples collected from multiple locations before DNA extraction. A strategy of pooling of eDNA samples has been used commonly to reduce sample numbers for evaluations of microbial community structure^39–41^. Importantly, pooling eDNA could dilute DNA of rare species, resulting in masking a significant portion of communities if communities are extremely complex (e.g., soil microbial community)^40^. However, communities of aquatic macroorganisms are obviously much less complex than microbial communities in soil and, thus we hypothesized that pooling of eDNA samples potentially might be effective to assess biodiversity of freshwater fishes.

Accordingly, we investigated the community composition of freshwater fishes in the satellite lakes of Lake Biwa, Japan, using eDNA metabarcoding. By comparing detected fish communities between the individual and pooled samples taken from the same sites, we tested whether pooling of eDNA samples (pooling of water samples) could reduce the effectiveness of eDNA metabarcoding with respect to the biodiversity assessment.

## Results

### MiSeq sequencing and taxon assignment

In total, 9,083,566 MiSeq reads were obtained, of which 8,139,675 passed the quality control processes (Supplementary Table S1). Of these reads, 84.8% (6,801,895 reads) were more than or equal to 10 reads, and they were clustered into 29,868 unique sequences. Among these unique sequences, 24,192 were successfully assigned to 50 lineages. Sequences of fish lineages were not found in the negative controls. After removing seven fish lineages that are unlikely to inhabit the study areas (Supplementary Table S2), the remaining 43 lineages were subjected to the subsequent analyses (Supplementary Table S3).

### Spatial signals of fish communities detected in individual samples

**Figure 1:**
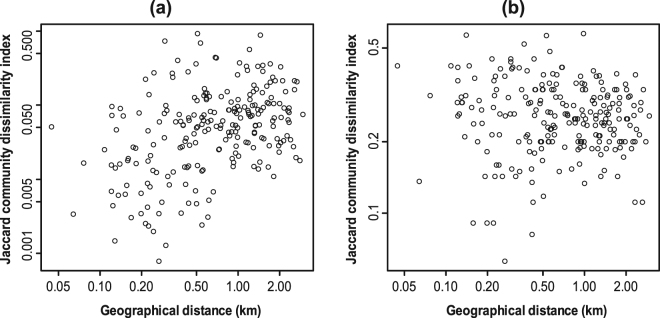
Euclidean geographic distances among sampling locations plotted against abundance-based (**a**) and incidence-based (**b**) Jaccard community dissimilarity indices of fish lineages.

The Adonis test for the individual samples indicated that geographic locations (latitudes) of water sampling had a significant effect on the abundance-based and incidence-based Jaccard community dissimilarities (Table 1), indicating positive spatial autocorrelation. Less distinctly, water pH was correlated with the abundance-based dissimilarity index (Table 1). Results of the Mantel test (Fig. 1) showed that geographic distances among sampling locations had a significantly positive correlation with the abundance-based community dissimilarity index (Mantel *r* = 0.329, *P* = 0.008), but the correlation was not significant in the incidence-based index (Mantel *r* = 0.303, *P* = 0.528).

**Table 1:** Adonis test for the effects of geographic locations and environmental factors, including water temperature, pH, and EC, on fish community structure at each satellite lake.

Community dissimilarity index	Variable	R^2^	P
Abundance-based Jaccard index	Latitude	0.547	0.012
	pH	0.146	0.012
	EC	0.055	0.053
	Temperature	0.047	0.127
	Residuals	0.204	
	Total	1.000	
Incidence-based Jaccard index	Latitude	0.149	0.010
	pH	0.033	0.069
	EC	0.024	0.788
	Temp	0.069	0.074
	Residuals	0.725	
	Total	1.000

### Species richness observed in individual and pooled samples

**Figure 2:**
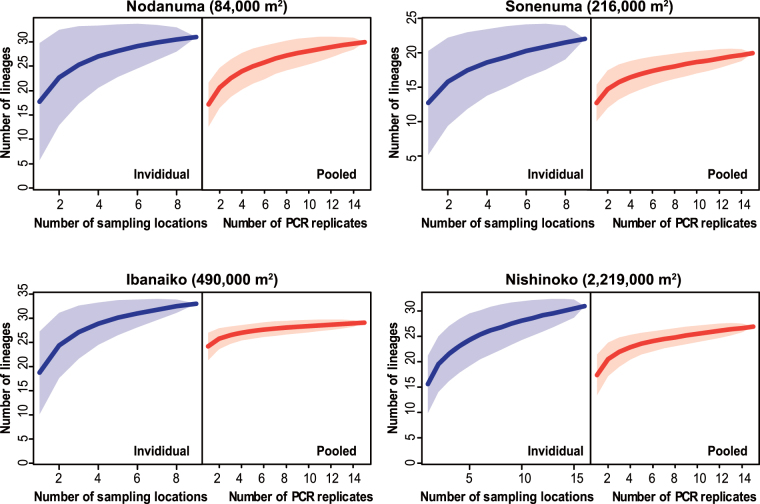
Accumulation curves of fish lineages (proxies for fish species) at each satellite lake with increasing number of sampling locations for the individual samples (blue line) and increasing number of PCR replicates for the pooled samples (red line). The shaded area represents 95% confidence intervals of the accumulation curve.

**Figure 3:**
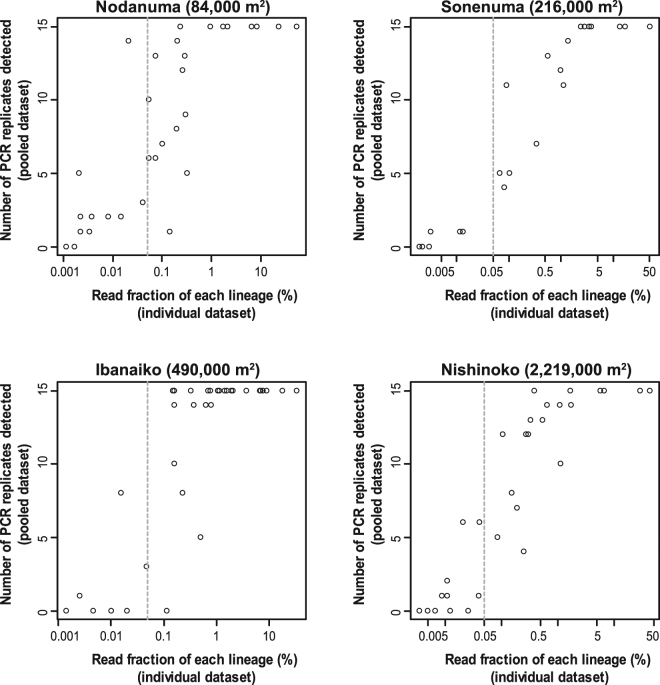
Read fraction of respective fish lineages in individual samples of each satellite lake plotted against number of PCR replicates in the pooled samples where sequences of the same lineages are detected. Each point represents a fish lineage detected. A vertical dashed line represents an approximate threshold value (0.05%) below which fish lineages are mostly undetectable in the pooled samples.

In the individual samples, the total numbers of lineages detected from the Nodanuma, Sonenuma, Ibanaiko, and Nishinoko sites were 31, 22, 33, and 31, respectively. In the pooled samples, 30, 20, 29, and 27 lineages were observed from each of the four satellite lakes, respectively, with 15 PCR replicates. The number of lineages that were detected in the individual sample but not in the pooled sample increased with increasing the surface area of satellite lake (Supplementary Fig. S1). Moreover, the number of lineages increased with sampling effort either by adding sampling locations for the individual samples or alternatively, by increasing PCR replicates for the pooled samples (Fig. 2). Nevertheless, the species accumulation curve for each site nearly reached an asymptote (Fig. 2; Supplementary Fig. S2). For every satellite lake, the number of lineages estimated with the Jack1 estimator for the individual samples was larger than that estimated for the pooled samples (Table 2). Lineages that accounted for less than 0.05% of the total read count of each site’s individual samples were mostly undetectable in PCR replicates of the pooled samples (Fig. 3).

**Table 2:** Number of observed and estimated lineages for each satellite lake.

Satellite lake	Data source	Samples	Lineages observed	Jack1 (SE)
Nodanuma	Individual	9	31	35.44 (3.82)
	Pooled	15	30	33.73 (2.31)
Sonenuma	Individual	9	22	26.44 (3.82)
	Pooled	15	20	23.73 (1.87)
Ibanaiko	Individual	9	33	37.44 (2.39)
	Pooled	15	29	30.87 (1.32)
Nishinoko	Individual	16	31	37.56 (3.69)
	Pooled	15	27	30.73 (1.87)

Number of lineages (proxies for species) at each site is estimated using the nonparametric first-order jackknife (Jack1) estimator. These numbers are summarized according to the data sources (the individual versus pooled samples).

### Fish communities detected in individual and pooled samples

**Figure 4:**
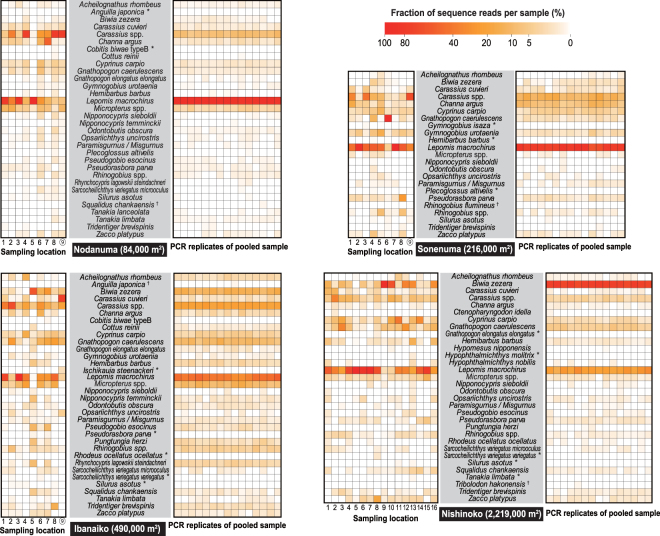
Heatmaps depicting a relative read fraction of each lineage per sample. Results of the individual and pooled samples are shown on left and right sides, respectively. Asterisks and daggers indicate lineages detected only in the individual and pooled samples, respectively. Sampling locations around the center of the satellite lakes are indicated by encircled numbers.

Among 43 fish lineages detected, *Lepomis macrochirus* (exotic bluegill) showed the most abundant sequence reads in most of the samples (Fig. 4). Although showing slightly less abundant sequence reads, *Carassius* spp. and *Cyprinus carpio* were detected in all samples (Fig. 4). *Gnathopogon caerulescens* and *Micropterus* spp. (species complex of exotic *Micropterus salmoides* and *Micropterus floridanus*) were also frequent and were characterized by relatively abundant reads, but the latter was relatively rare in the Sonenuma samples (Fig. 4). *Biwia zezera* was characterized by relatively high frequency and abundant reads in the Ibanaiko and Nishinoko samples (Fig. 4). The majority of the lineages detected around the center of the satellite lakes were also detected around the shore, but, exceptionally, *Ischikauia steenackeri* was found solely in the center of Ibanaiko (Fig. 4; Supplementary Table S3).

The majority of the lineages were detected in the individual and pooled samples from all the satellite lakes. Numbers of lineages that were not found in the pooled samples but were found in the individual samples were two (*Anguilla japonica* and *Cobitis biwae* type B), three (*G*. *isaza*, *Hemibarbus barbus*, and *Plecoglossus altivelis*), five (*I*. *steenackeri*, *Pseudorasbora parva*, *Rhodeus ocellatus ocellatus*, *Sarcocheilichthys variegatus variegatus*, and *Silurus asotus*), and five (*Gnathopogon elongatus elongatus*, *H*. *molitrix*, *S*. *variegatus variegatus*, and *S*. *asotus*, *Tanakia limbata*) for the Nodanuma, Sonenuma, Ibanaiko, and Nishinoko sites, respectively (Fig. 4). Moreover, *Squalidus chankaensis*, *Rhinogobius flumineus*, *Anguilla japonica*, and *Tribolodon hakonensis* were not observed in the individual samples but were observed in the pooled samples of the Nodanuma, Sonenuma, Ibanaiko, and Nishinoko sites, respectively (Fig. 4).

**Figure 5:**
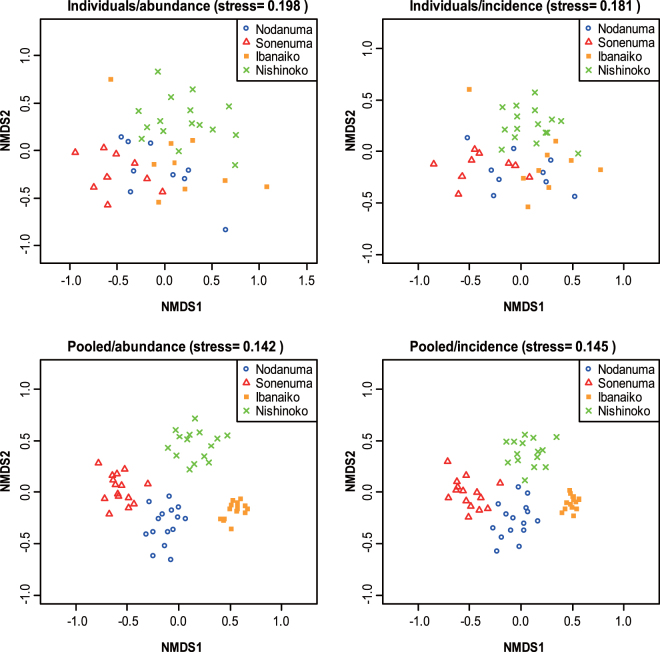
Two-dimensional NMDS plot of fish community in each satellite lake. Upper and lower sides represent the NMDS plot of the individual and the pooled samples, respectively. The NMDS plots are performed based on abundance-based (left) and incidence-based (right) Jaccard community dissimilarity indices, separately. Smaller stress values (e.g., those less than 0.2) indicates better fits of two-dimensional plotting to the original data.

Moreover, the relative read fractions of respective lineages were considerably different among samples within the individual samples, whereas they appeared to be highly similar among samples within the pooled samples (Fig. 4). Similarly, results of the NMDS indicated that the fish communities were substantially variable among different sampling locations within the same satellite lake (the individual samples), whereas the community compositions were similar among PCR replicates of the same satellite lakes in the pooled samples (Fig. 5). These results were mostly consistent between the abundance-based and incidence-based community dissimilarity indices (Fig. 5). Moreover, the PERMANOVA analysis suggested that the community structure varied between the individual and pooled samples (abundance-based, *R* ^2^ = 0.115, *P* = 0.0126; incidence-based, *R* ^2^ = 0.030, *P* = 0.0001). The subsequent PERMDISP analysis further indicated that among-sample heterogeneity of fish communities within the same satellite lake was significantly different between the individual and pooled samples (abundance-based, *P* = 0.001; incidence-based, *P* = 0.0490).

## Discussion

We present the first attempt to compare the effectiveness of individual versus pooled samples to assess biodiversity of fish communities in freshwater using eDNA. Based on the results, we discuss potential limitations and applications of the pooling method for assessing biodiversity using eDNA metabarcoding.

### Properties of individual samples and spatial autocorrelation

The fish communities observed in the individual eDNA samples have implications for understanding spatial structure of eDNA in the satellite lakes. Our results indicated that increasing the number and spatial replication of samples leads to increasing sensitivity and reliability of estimating species richness of fishes in the satellite lakes using eDNA metabarcoding (Fig. 2). Importantly, fish communities detected in the eDNA samples appeared to be moderately spatially autocorrelated in the satellite lakes (Table 1; Fig. 1). These findings are similar to those of previous studies^31,33,36^, suggesting that not only sampling volume but also geographic distance between sampling locations are essential considerations for assessing biodiversity using eDNA metabarcoding. Although the spatial signals were somewhat obscured in the incidence samples (Table 1; Fig. 1), this is presumably due to the lack of read depth information, which is roughly correlated with the concentration gradient of eDNA. Moreover, sampling of water in the center may not greatly improve the detection rate of fish communities at our sampling scales (Fig. 4). The present findings suggested that detectability of eDNA metabarcoding can be improved by collecting a small amount of water from many locations. The detectability would be also increased by collecting a large amount of water from a single location, but this sampling strategy seems ineffective to address spatial autocorrelation^42^. Nevertheless, the latter sampling strategy might be applicable to small areas (e.g., Nodanuma in this study, the surface area of which is 84,000 m^2^), as reported previously^38^.

### Limitations of use of pooling samples for eDNA metabarcoding

We demonstrated the limitations of a pooling strategy for assessing biodiversity of freshwater fish communities by eDNA metabarcoding. The results indicated that use of a pooling strategy leads not only to saving labor but also to missed detection of fish lineages that were detected in the individual samples, resulting in a slight decrease in detection rates in eDNA metabarcoding (Table 2; Figs 2–4). This is especially true for the satellite lakes with large surface areas (e.g., Nishinoko), in which the spatial autocorrelation of community composition was likely distinct. Missed detection of fishes can be primarily attributed to the stochastic loss of rare fish lineages that can be caused by a limited volume of filtered water in pooled samples, as cautioned previously^4,24,28,43^. Filtering a double water volume of the pooled samples slightly improved the detection rates (pooled-1, 2 [filtration of 500 ml] vs. pooled-3 [filtration of 1000 ml] in Supplementary Fig. S2) and, thus, stochastic loss of rare lineages might not be reduced unless rendering a filtration volume almost equivalent to the sum of that of the individual samples. In addition, an insufficient number of PCR replicates may cause serious underestimation of biodiversity^4,44,45^. This concern may be relevant especially to the pooled samples, for which spatially autocorrelated (thus, heterogeneous) fish communities were combined. However, the number of fish lineages detected in the pooled samples nearly reached an asymptote with increasing number of PCR replicates (Fig. 2) and, thus, the stochastic loss would account more reasonably for decreased detection rates in the pooled samples. Both of these effects appeared to influence not only the pooled samples but also the individual samples (indeed, a few fish lineages were detected solely in the pooled samples; Fig. 4), but the potential bias would be more serious in the former samples. These results suggested that pooling of spatially autocorrelated samples for eDNA metabarcoding is a labor-saving method but it is unsuitable for assessing species richness and alpha diversity of fish species in the underlying sites, regardless of the size of site, similar to the case of soil microbial community^40,46^. For these purposes, although more labor is required, we recommend the use of a sufficient number of individual samples.

Not considering the efficiency, several alternative strategies are possible to reduce the stochastic loss. For instance, the stochastic loss will be reduced by filtration of the entire volume of the pooled water. To do this, however, there is a need to replace the sample filters again and again to prevent clogging. Another procedure is pooling of extracted template DNA instead of collected water samples, but this procedure is obviously inefficient because of an increase of labor burdens in the molecular experiments (DNA extraction and PCR). Because the major advantage of the pooling strategy is its efficiency, we do not suggest use of these alternative procedures.

False-positives are also more likely in pooled samples because greater numbers of PCR replicates increase PCR-induced artifacts and opportunities for contamination^45^. However, the risk of false-positives in the pooled samples seems to be similar to that in the individual samples in the present study, in which fish communities detected in the pooled samples were almost nested in those detected in the individual samples.

### Potential applications of use of pooling samples for eDNA metabarcoding

Despite its limitations, a sample pooling strategy (pooling of water) potentially serves to compare community structure (beta diversity) of fishes among sites. Small among-sample heterogeneity of fish communities detected in the pooled samples (Figs 4 and 5; analyses of PERMANOVA and PERMDISP) suggests that a pooling-sampling approach may allow to minimize between-sampling-location-variability and thereby to estimate optimal representation of fish communities, as similar to soil microbial communities^39,40,46^. Although locally dominant but spatially rare phylotypes often become undetectable in the pooled samples for soil microbial communities^40^, such trends do not appear to be true for the present data (Figs 3 and 4). This is probably due to a moderate alpha- and beta-diversity of freshwater fishes comparing soil microbes^47,48^. Therefore, negative effects are less pronounced for studies of freshwater fish compared to microbes. These findings suggested that the pooling strategy in eDNA metabarcoding is potentially useful for among-site comparison of representative communities of freshwater fishes and other aquatic vertebrates.

However, caution must be taken when using pooling strategy for eDNA metabarcoding. Firstly, use of the pooling method would increase the risk of false-negatives (PCR dropouts). Therefore, a sufficient number of PCR replicates (e.g., ≥8 replicates, as suggested previously^45^) is presumably crucial for this method, although the number of PCR replicates should not be increased unlimitedly to avoid the potential risk of false-positives^45^. Moreover, rates of dropouts in the pooled samples appeared to increase with increasing the surface areas of satellite lakes (Table 2; Fig. 2), suggesting that pooling of highly heterogeneous fish communities may lead to missed detection of a large portion of rare species, similar to the case of microbial communities^40,46^. Therefore, we suggest the pooling should be restricted to samples that are collected from spatially adjacent and environmentally similar areas, as in the case of the present study.

There is increasingly a need to monitor large-scale spatiotemporal variations of the community structure of aquatic organisms using eDNA metabarcoding^29,36^. The pooling procedure as used in the present study is presumably one of the practical strategies for large-scale surveys of aquatic organisms using eDNA metabarcoding.

## Conclusion

Our results suggested that detectability of freshwater fish lineages in eDNA metabarcoding is increased by collecting a small amount of water from many locations. By comparing the number of freshwater fishes detected in the satellite lakes between individual and pooled samples in eDNA metabarcoding, we demonstrated that pooling of spatially autocorrelated samples likely leads not only to saving labor but also to somewhat underrepresentation of fish lineages, especially in the satellite lakes with large surface areas. Therefore, a pooling procedure for eDNA metabarcoding should not be used to assess species richness and alpha diversity of freshwater fishes. Nevertheless, the pooling strategy appears to minimize between-sampling-location variability and thereby allow for estimating representation of fish communities. Although caution is required, the pooling strategy in eDNA metabarcoding is potentially useful for among-site comparison of representative communities of freshwater fishes and other aquatic vertebrates.

## Materials and Methods

### Research site, Water sampling and field survey

**Figure 6:**
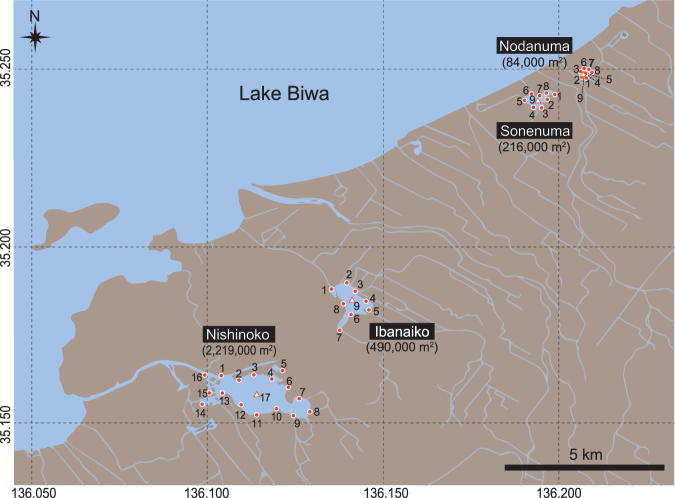
Map of the study sites (Nodanuma, Sonenuma, Ibanaiko, and Nishinoko) with locations of water sampling for metabarcoding. Sampling locations near shore are shown as closed circles, whereas those around the center of the satellite lakes are shown as open triangles. More detailed information for each sampling location is provided in Supplementary Table S4. This map was created using QGIS version 2.14 (QGIS Development Team, 2016. QGIS Geographic Information System. Open Source Geospatial Foundation Project. http://www.qgis.org/) based on OpenStreetMap. The cartography in the OpenStreetMap map tiles is licensed under CC BY-SA (www.openstreetmap.org/copyright, © OpenStreetMap contributor). The license terms can be found on the following link: http://creativecommons.org/licenses/by-sa/2.0/.

Water samples for eDNA analyses were collected from the surface layer of four satellite lakes (Nodanuma, Sonenuma, Ibanaiko, and Nishinoko; the surface areas are 84,000, 216,000, 490,000, and 2,219,000 m^2^, respectively; the depth of water ranges from 1 to 2 m) of Lake Biwa in Shiga Prefecture, Japan. Sampling locations from each satellite lake were located around the edges of the lakes, with one in the center (Fig. 6; 17 positions for Nishinoko, and 9 positions for Nodanuma, Sonenuma, and Ibanaiko, respectively). Sampling locations in the center of satellite lakes in Nodanuma, Sonenuma, Ibanaiko, and Nishinoko were approximately 65 m, 130 m, 230 m, and 580 m away from the shorelines. All sampling devices were washed with a bleach solution before use. Approximately 500- and 250-ml water samples were collected separately from the water surface at each sampling location using plastic beakers with a handle. Each of the 500-ml water samples (hereafter, “individual samples”) was filtered immediately through a GF/F glass fiber filter (nominal pore size = 0.7 μm; diameter = 47 mm; GE Healthcare Japan Corporation, Tokyo, Japan). The 250-ml water samples collected from the same satellite lakes were pooled into a plastic tank (resulting in 4250 ml water for Nishinoko and 2250 ml water for another satellite lake). The entire tank of water could not be collected with a single filter because of clogging, and, thus, 500 ml of the tank water was filtered to avoid clogging and to save labor in this step (hereafter, “the pooled samples”). One pooled sample was prepared for each of the Nodanuma, Sonenuma, and Ibanaiko sites. For Nishinoko, three pooled samples were prepared, for one of which the filtration volume was doubled (to prevent clogging, it was divided into two equal volumes, which were filtered separately). One negative control sample was also taken by filtering 500 ml of Milli-Q water at each of the four satellite lakes to monitor contamination during the filtering and subsequent DNA extraction. Each filter was folded in half using tweezers with the filter surface on the inside of the fold, wrapped in aluminum foil, placed in a plastic bag, and then stored at −20◦C before the subsequent DNA extraction process.

In addition, water temperature, pH and electrical conductivity (EC) of the “individual” water samples were measured in the field using portable waterproof testers (Waterproof EC/TDS/Temperature Testers, Hanna Instruments, Maurituis; Supplementary Table S4).

### DNA extraction, Paired-end library preparation, MiSeq sequencing

Total eDNA was extracted from each filter using a DNeasy Blood and Tissue Kit (Qiagen, Hilden, Germany) in combination with a spin column (EZ-10; Bio Basic, Markham, Ontario, Canada). After removing the originally attached membrane of the spin column (EZ-10), the sample filter was placed in the spin column (EZ-10). The spin column with the sample filter was centrifuged at 6000 g for 1 minute and water remaining in the sample filter was removed (the filtration was discarded). After placing the spin column in a new 2-mL tube, the mixed solution of Milli-Q water (200 μL), proteinase K (10 μL), and Buffer AL (100 μL) was pipetted gently onto the filter in the spin column. The tube was then incubated at 56◦C for 30 minutes. After incubation, the liquid held in the filter was collected by centrifugation. To increase the yield of eDNA, 200 μL TE buffer was pipetted gently onto the filter and the spin column was centrifuged again at 6000 g for 1 minute. The collected DNA solution was purified using the DNeasy Blood and Tissue Kit following the manufacture’s protocol, except for a modification in the final step of elution of DNA from the filter column. We eluted DNA using 100 μL Buffer AE, though the original manual specified 200 μL.

A two-step PCR-procedure was used for library preparation. In the first step, a fragment of the mitochondrial 12S rRNA gene was amplified using the MiFish-U-F and MiFish-U-R primers^21^, which were designed to contain Illumina sequencing primer regions and 6-mer Ns for improved“chastity”in Illumina sequencing (forward: 5′-ACACTCTTTCCCTACACGACGCTCTTCCGATCTNNNNNN GTCGGTAAAACTCGTGCCAGC-3′, reverse: 5′-GTGACTGGAGTTCAGACGTGTGCTCTTCCGATCTNNNNNN CATAGTGGGGTATCTAATCCCAGTTTG-3′), in which NNNNNN represents a 6–base pair (bp) random sequence. We used a KOD FX Neo polymerase (Toyobo, Osaka, Japan) for the first PCR to facilitate amplifications of DNA from crude extracts. The first PCR was performed with a 12 μL reaction volume containing 1 × PCR Buffer for KOD FX Neo, 0.4 mM dNTP mix, 0.24 U KOD FX Neo polymerase, and 3.5 pmol of each primer. The thermal cycles of this step were as follows: initial denaturation at 94 °C for 2 minutes, followed by 35 cycles of denaturation at 98 °C for 10 seconds, annealing at 65 °C for 30 seconds, and elongation at 68 °C for 30 seconds, followed by final elongation at 68 °C for 5 minutes. The first PCR was replicated 3 and 15 times per sample for the individual and pooled samples, respectively. The PCR replicates of the individual samples were pooled to mitigate the false negatives (PCR dropouts), whereas those of the pooled samples were subjected to the second PCR, separately. The first PCR products were purified using Exo-SAPIT (Affymetrix, Santa Clara, CA, USA) according to the manufacturer’s instructions. The purified first PCR products were used as templates for the second PCR.

The Illumina sequencing adaptors plus the 8-bp identifier indices^49^ were added in the subsequent PCR process using a forward and reverse fusion primer (forward: 5′-AATGATACGGCGACCACCGAGATCTACA - index-ACACTCTTTCCCTACACGACGCTCTTCCGATCT-3′, reverse: 5′-CAAGCAGAAGACGGCATACGAGAT-index-GTGACTGGAGTTCAGACGTGTGCTCTTCCGATCT-3′). The second step was done with a 12 μL reaction mixture containing 1 × KAPA HiFi HotStart ReadyMix (KAPA Biosystems, Wilmington, WA, USA), 3.5 pmol of each primer, and 1 μL of the PCR products. The thermal cycles of the second PCR were as follows: initial denaturation at 95 °C for 3 minutes, followed by eight cycles of denaturation at 98 °C for 20 seconds, annealing and elongation combined at 72 °C for 15 seconds, with a final elongation at 72 °C for 5 minutes.

The indexed second PCR products were pooled in equal volumes and 25 μL of the pooled libraries were loaded on a 2% E-Gel SizeSelect (Thermo Fisher Scientific, Waltham, MA, USA) and a target size of the libraries (ca. 370 bp) was collected. The DNA concentrations were then estimated by a Qubit dsDNA HS assay kit and a Qubit fluorometer (Thermo Fisher Scientific). The amplicon libraries were sequenced by 2 × 150 bp paired-end sequencing on the MiSeq platform using the MiSeq v2 Reagent Kit according to the manufacturer’s instructions.

### Sequence read processing, taxonomic assignment, and preparation of community data

The overall quality of the obtained sequence (deposited in the DDBJ Sequence Read Archive, BioProject accession: PSUB007167) was evaluated by the program FASTQC (available from http://www.bioinformatics.babraham.ac.uk/projects/fastqc/). After trimming low-quality tails from each read using DynamicTrim.pl from the SOLEXAQA software package^50^ with a cutoff threshold set at a Phred score of 10, the paired-end reads were assembled using the software FLASH with a minimum overlap of 10 bp^51^. The assembled reads were filtered further to remove forward and reverse primer positions, ambiguous sites (Ns), and sequences showing unusual lengths.

The preprocessed reads from the above custom pipeline were dereplicated using UCLUST, with the number of identical reads added to the header line of the FASTA formatted data file. The sequences represented by more than or equal to 10 identical reads were subjected to the downstream analyses and the remaining under-represented sequences (with less than 10 identical reads) were subjected to pairwise alignment using a “usearch global” command in UCLUST. If the latter sequences showed more than or equal to 99% identity with one of the former reads, they were considered operationally as identical and they were merged.

The processed reads were subjected to local BLAST searches^52^ against the comprehensive reference database of fish species that were established previously^21^, using an e-value cutoff of 10^−5^ and an identity cutoff of 99%. This procedure also works to removing erroneous reads because erroneous reads are expected never to match the reference sequences at ≥99% similarity by chance. Because some closely-related species shared identical sequences in the barcode region, those species were merged (i.e., treated as species complex) before the BLAST searches (Supplementary Table S5). In addition, species that are unlikely to inhabit the study areas (e.g., marine species) were removed from the BLAST hits (listed in Supplementary Table S2). The BLAST top hits (those with the highest identity with query sequence) were then applied to species assignments of each representative sequence. The majority of the representative sequences were assigned to single taxa, except for five sequences that had a single base mismatch with either of those of two taxa (i.e., *Carassius* spp. vs. *Carassius cuvieri* or *Carassius* spp. vs. *Cyprinus carpio*). These exceptional sequences were not included in the downstream analyses because they accounted for a small proportion of the total reads (0.0137%) and taxonomic assignment of them did not influence the presence/absence of fishes in each sample. After BLAST searches, sequences that were assigned to the same species (or species complex) were clustered, and we considered the clustered sequences as proxies for species (hereafter, called “lineages”). Because most of the sequence reads detected in 17th sample of the individual samples from the Nishinoko site (i.e., the sample collected at the center of the satellite lake) failed to be assigned to a lineage name, presumably due to failure in the PCR step, this sample was not used for the subsequent analyses.

The sequencing reads of respective fish species were recorded for each sample and these data were arranged into a matrix in which the rows and columns represent sample IDs and fish species, respectively. Before the community-based analyses, we confirmed that the sequencing depth was sufficient to detect the α-diversity perfectly in each sample with the function “rarecurve” as implemented in the vegan v.2.4-2 package^53^ of R 3.1.2^54^ (shown in Supplementary Fig. S3).

In addition, the number of fish lineages and composition of fish communities were almost identical among the aforementioned three pooled samples for the Nishinoko site (Supplementary Fig. S2) and, thus, hereafter for simplicity we used only the first sample (500 ml water was filtered for this sample) for the purpose of comparing results of the individual and pooled samples.

### Spatial and environmental variations of fish community

Firstly, we measured the spatial autocorrelation of fish communities in the individual samples. We evaluated the relative importance of geographic locations within each satellite lake (latitude and longitude) and water environmental variables (water temperature, pH, and EC) on the composition of fish species (a dataset of the individual samples), with the “adonis” function of the vegan package, in which permutations were constrained within each satellite lake. For this analysis, single factor models were first tested and, thereafter, factors were added in the final model in order of their *R* ^2^ values. Since the latitude and longitude were highly correlated with each other (Pearson’s coefficient, *r* = 0.981), only the latitude was included in the model.

A Mantel test was also performed to test whether fish communities were clustered by geographic locations (“‘mantel” function of the vegan). For the analysis, permutations were constrained within each satellite lake using the “strata” argument. The Mantel test *R* statistic indicates the Pearson’s correlation between the multivariate fish community structures detected in samples and the geographic distance between samples.

### Comparison of detectability of fish community between the individual and pooled samples

We compared the number of lineages detected in the individual samples to that in pooled samples. Sample-based species accumulation curves were calculated based on the sample-species matrix using the “specaccum” command in the vegan package of R, thereby testing whether numbers of sampling locations (the individual samples) and PCR replicates (the pooled samples) were large enough to allow measurement of species richness at each site using sample-based species accumulation curves. We also estimated the total number of lineages at each site using nonparametric first-order jackknife (Jack1) estimator and confidence interval (Jack1 ± 2 SE), implemented in the “specpool” function of the vegan. Moreover, the read fractions of respective fish lineages in each individual sample were plotted against the number of samples (PCR replicates) in the pooled samples where sequences of the same lineages were detected, to identify fish lineages that were detectable only in the individual samples.

We also compared the fish community detected in eDNA metabarcoding between two sources (the individual vs. the pooled samples). Primarily, the relative read fraction of individual fish lineage within each sample was converted into a heatmap using the function “pheatmap” as implemented in pheatmap v.1.0.8 package of R. In addition, differences in the community compositions were visualized using the two-dimensional nonmetric multidimensional scaling (NMDS) ordination with “metaMDS” function in the vegan package of R, where the program choose the best solution (i.e., solution with the lowest stress value) from 100 separate runs of real data. For the NMDS, the community dissimilarity was calculated based on abundance-based (use of sequence reads) and incidence-based (presence/absence) Jaccard indices.

We tested for variability in the community composition between two sources using a permutational multivariate analysis of variance (PERMANOVA; “adonis” function of the vegan package) with 9999 permutations, based on abundance-based and incidence-based Jaccard dissimilarity indices. We also tested heterogeneity of dispersion between sources using a permutational analysis of multivariate dispersion (PERMDISP; “betadisper” function of the vegan package). For those statistical analyses, permutations were constrained within each satellite-lake using the “strata” argument to account for nestedness.

## Electronic supplementary material

Supporting information
