# Supplementary material for: Usefulness and limitations of sample pooling for environmental DNA metabarcoding of freshwater fish communities
Source: Sci Rep. 2017 Nov 1;7:14860. doi: 10.1038/s41598-017-14978-6 (PMC5665893; doi:10.1038/s41598-017-14978-6)
Supplement: Supplementary file 1 — Supporting information [file 41598_2017_14978_MOESM1_ESM.pdf]

Usefulness and limitations of sample pooling for environmental DNA metabarcoding of freshwater fish communities

Hirotoshi Sato[1*], Yuki Sogo[1], Hideyuki Doi[2], Hiroki Yamanaka[1]

1. Department of Environmental Solution Technology, Facility of Science & Technology, Ryukoku University, Seta-Oe, Otsu, 520-2194 Shiga, Japan
2. Graduate School of Simulation Studies, University of Hyogo, Minatojima-minamimachi, Kobe, 650-0047, Japan

E-mail (corresponding author): h-sato@sys.bot.kyoto-u.ac.jp (HS)

This file contains three Supplementary Figures and five Supplementary Tables.

Fig. S1. Decrease of detection rates of fish lineages in pooled samples with increasing surface areas of satellite lakes.

Fig S2. (a) Accumulation curves of fish lineages (proxies for fish species) with increasing number of PCR replicates, and (b) heatmaps depicting a read fraction of each lineage per sample, for three pooled samples for Nishinoko.

Fig. S3. Accumulation curves of fish lineages (proxies for fish species) with increasing total sequence reads per sample.

Table S1. Number of reads passed through each quality control process.

Table S2. List of fish species that are removed prior the community analyses.

Table S3. A data matrix depicting sequence reads of respective fish lineages in each sample.

Table S4. Geographical locations and environmental conditions of respective sampling locations.

Table S5. The united lineage name for species that shared identical sequences.

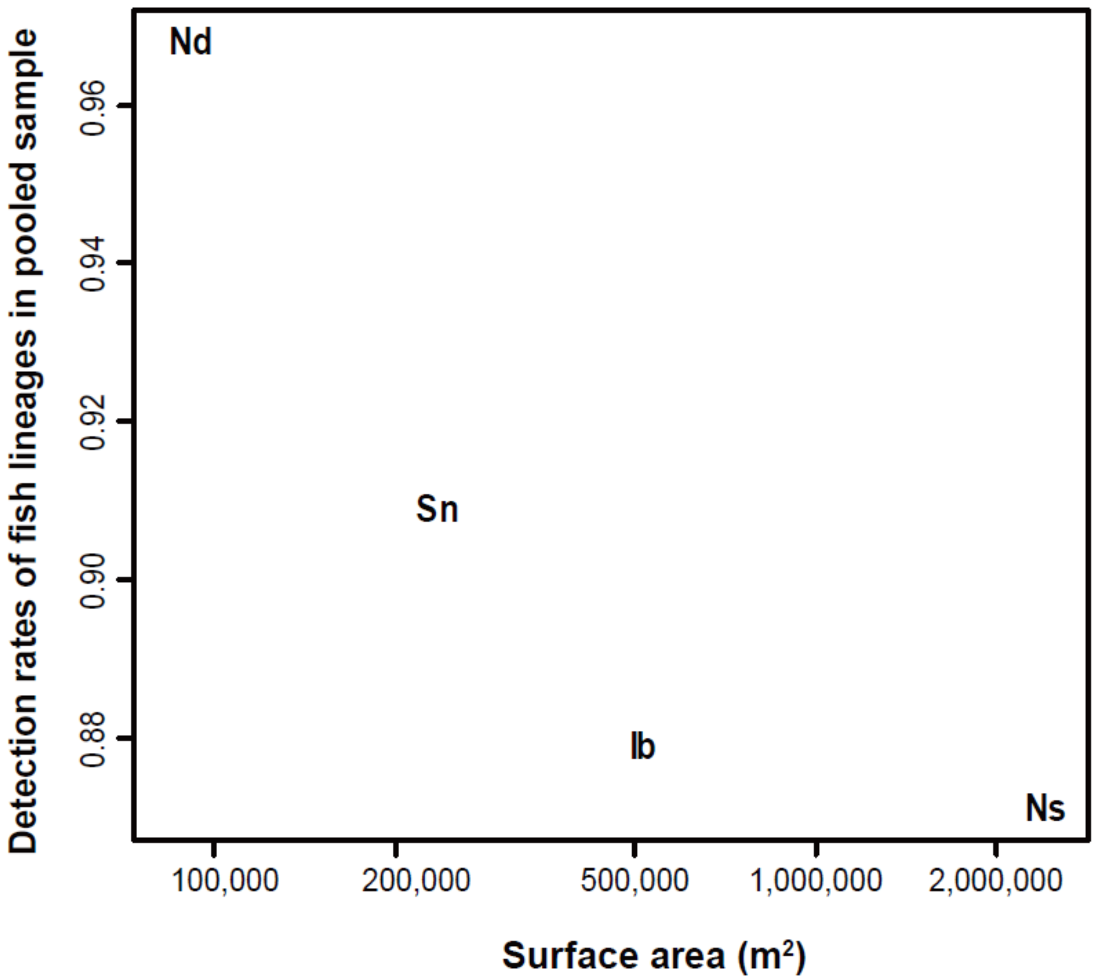

Fig S2. Decrease of detection rates of fish lineages in pooled samples with increasing surface areas of satellite lakes. The vertical axis represents the number of fish lineages detected in the pooled sample divided by that detected in the individual sample. Two characters abbreviation represents the names of satellite lakes (Nd: Nodanuma, Sn: Sonenuma, Ib: Ibanaiko, and Ns: Nishinoko).

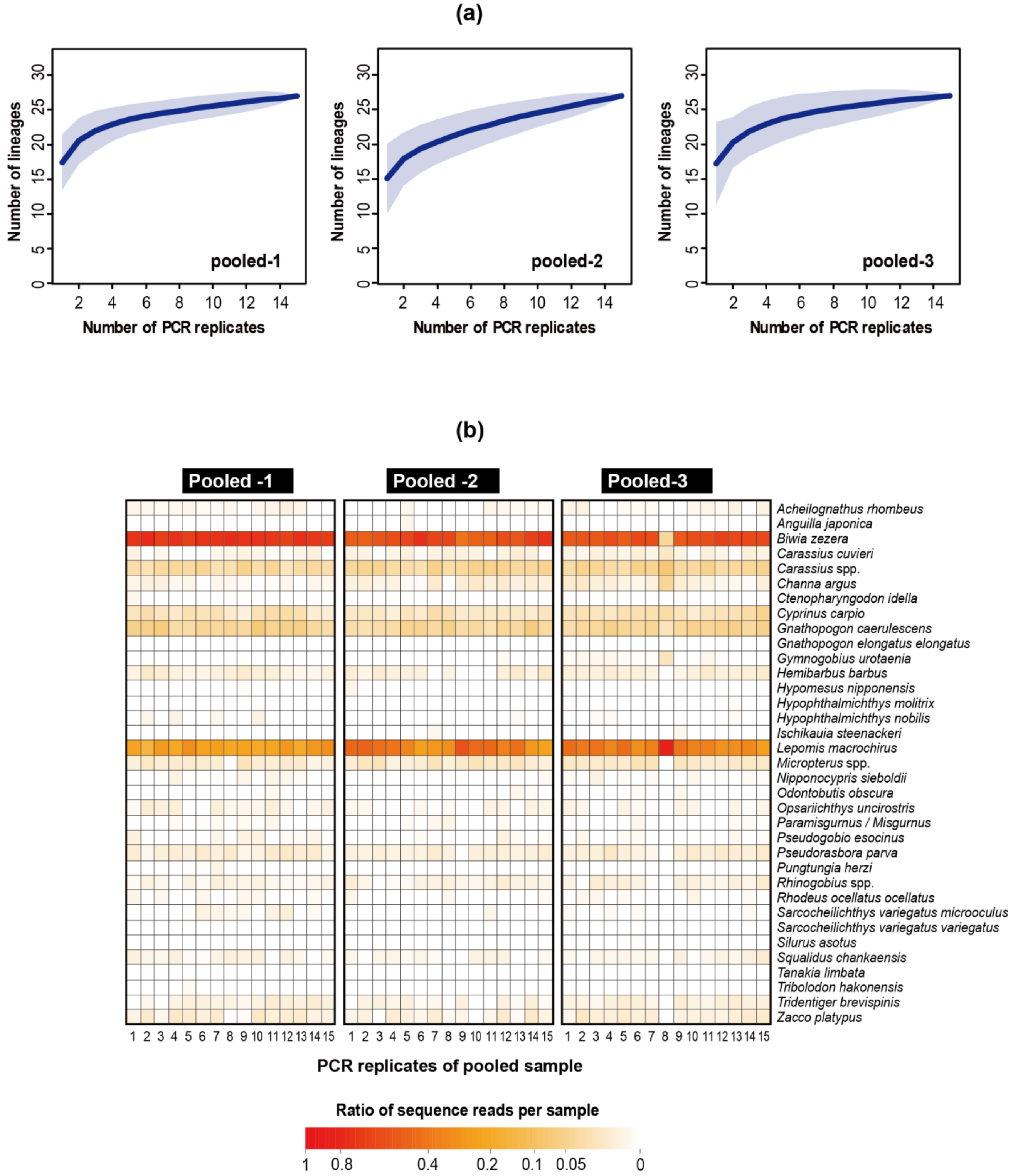

Fig S2. (a) Accumulation curves of fish lineages (proxies for fish species) with increasing number of PCR replicates, and (b) heatmaps depicting a read fraction of each lineage per sample, for three pooled samples for Nishinoko. Only for the pooled-3 sample, the filtration volume was doubled.

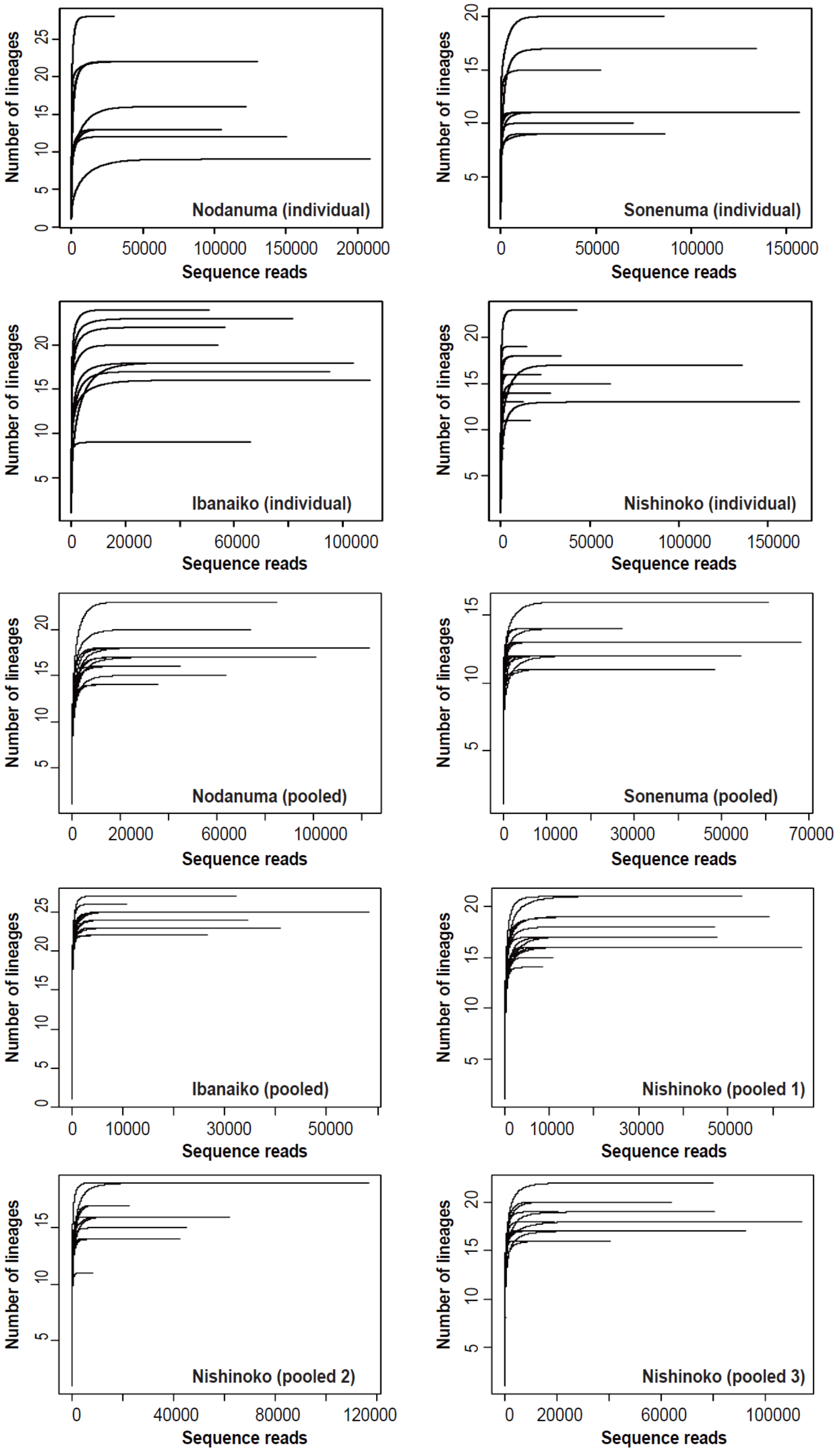

Fig. S3. Accumulation curves of fish lineages (proxies for fish species) with increasing total sequence reads per sample. For all samples, α-diversity perfectly reached saturation in each sample.

Table S1. Number of reads passed through each quality control process.

Sample name	Raw	Assembled (%)	Length-filtered (%)	Primer-removed (%)	≥10 reads (%)	Lineage assigned (%)
ND_individual_1	143292	141253 (98.6%)	140799 (98.3%)	140602 (98.1%)	125946 (87.9%)	122103 (85.2%)
ND_individual_2	97643	95731 (98.0%)	93772 (96.0%)	93644 (95.9%)	80302 (82.2%)	75958 (77.8%)
ND_individual_3	174048	170985 (98.2%)	170484 (98.0%)	170242 (97.8%)	153961 (88.5%)	150617 (86.5%)
ND_individual_4	105708	101902 (96.4%)	100741 (95.3%)	100621 (95.2%)	83977 (79.4%)	76436 (72.3%)
ND_individual_5	229983	226716 (98.6%)	225728 (98.1%)	225319 (98.0%)	211583 (92.0%)	209126 (90.9%)
ND_individual_6	163615	160199 (97.9%)	160097 (97.8%)	159908 (97.7%)	136973 (83.7%)	130413 (79.7%)
ND_individual_7	131357	128922 (98.1%)	127568 (97.1%)	127428 (97.0%)	110291 (84.0%)	104953 (79.9%)
ND_individual_8	61790	57086 (92.4%)	43521 (70.4%)	43454 (70.3%)	33332 (53.9%)	30141 (48.8%)
ND_individual_9	92575	89452 (96.6%)	88078 (95.1%)	87936 (95.0%)	70682 (76.4%)	62498 (67.5%)
SN_individual_1	141033	138533 (98.2%)	138069 (97.9%)	137835 (97.7%)	120067 (85.1%)	114497 (81.2%)
SN_individual_2	86656	84305 (97.3%)	79155 (91.3%)	79002 (91.2%)	70267 (81.1%)	69542 (80.3%)
SN_individual_3	114153	111067 (97.3%)	110004 (96.4%)	109827 (96.2%)	93235 (81.7%)	86029 (75.4%)
SN_individual_4	111142	108728 (97.8%)	105495 (94.9%)	105302 (94.7%)	87901 (79.1%)	85791 (77.2%)
SN_individual_5	66282	65054 (98.1%)	65019 (98.1%)	64898 (97.9%)	55042 (83.0%)	52619 (79.4%)
SN_individual_6	158638	155429 (98.0%)	154560 (97.4%)	154270 (97.2%)	136873 (86.3%)	133977 (84.5%)
SN_individual_7	177242	174650 (98.5%)	173947 (98.1%)	173615 (98.0%)	160322 (90.5%)	157004 (88.6%)
SN_individual_8	63152	61372 (97.2%)	57985 (91.8%)	57910 (91.7%)	48231 (76.4%)	47247 (74.8%)
SN_individual_9	62919	55793 (88.7%)	32579 (51.8%)	32505 (51.7%)	26783 (42.6%)	26276 (41.8%)

IB_individual_1	121044	117785 (97.3%)	117550 (97.1%)	117390 (97.0%)	100727 (83.2%)	95395 (78.8%)
IB_individual_2	80532	76916 (95.5%)	74514 (92.5%)	74412 (92.4%)	60326 (74.9%)	56644 (70.3%)
IB_individual_3	122433	120058 (98.1%)	118456 (96.8%)	118249 (96.6%)	106008 (86.6%)	104111 (85.0%)
IB_individual_4	144347	140079 (97.0%)	138872 (96.2%)	138655 (96.1%)	115425 (80.0%)	110361 (76.5%)
IB_individual_5	124610	119536 (95.9%)	104023 (83.5%)	103875 (83.4%)	83774 (67.2%)	81796 (65.6%)
IB_individual_6	74964	73279 (97.8%)	72345 (96.5%)	72255 (96.4%)	57846 (77.2%)	53984 (72.0%)
IB_individual_7	78924	74695 (94.6%)	65431 (82.9%)	65320 (82.8%)	56356 (71.4%)	50973 (64.6%)
IB_individual_8	98724	96470 (97.7%)	95393 (96.6%)	95252 (96.5%)	74934 (75.9%)	77596 (78.6%)
IB_individual_9	98000	89208 (91.0%)	80597 (82.2%)	80486 (82.1%)	68900 (70.3%)	65934 (67.3%)
NS_individual_1	59076	54090 (91.6%)	42380 (71.7%)	42327 (71.6%)	34371 (58.2%)	34065 (57.7%)
NS_individual_2	40663	34064 (83.8%)	13981 (34.4%)	13950 (34.3%)	10730 (26.4%)	10566 (26.0%)
NS_individual_3	55804	50759 (91.0%)	34923 (62.6%)	34861 (62.5%)	28786 (51.6%)	27744 (49.7%)
NS_individual_4	41153	37497 (91.1%)	27937 (67.9%)	27887 (67.8%)	22338 (54.3%)	22651 (55.0%)
NS_individual_5	190746	187529 (98.3%)	187383 (98.2%)	187102 (98.1%)	170907 (89.6%)	168116 (88.1%)
NS_individual_6	41865	36261 (86.6%)	15505 (37.0%)	15483 (37.0%)	12779 (30.5%)	12745 (30.4%)
NS_individual_7	53586	47115 (87.9%)	22498 (42.0%)	22467 (41.9%)	19052 (35.6%)	18967 (35.4%)
NS_individual_8	41886	36114 (86.2%)	15096 (36.0%)	15072 (36.0%)	11922 (28.5%)	11879 (28.4%)
NS_individual_9	161048	158240 (98.3%)	158066 (98.1%)	157826 (98.0%)	139785 (86.8%)	135927 (84.4%)
NS_individual_10	84493	81491 (96.4%)	77437 (91.6%)	77337 (91.5%)	62483 (74.0%)	61768 (73.1%)
NS_individual_11	45518	43660 (95.9%)	39666 (87.1%)	39595 (87.0%)	31490 (69.2%)	31085 (68.3%)

NS_individual_12	34434	32790 (95.2%)	27808 (80.8%)	27758 (80.6%)	22053 (64.0%)	21917 (63.6%)
NS_individual_13	63289	60307 (95.3%)	55255 (87.3%)	55149 (87.1%)	43183 (68.2%)	42599 (67.3%)
NS_individual_14	35367	30615 (86.6%)	12753 (36.1%)	12727 (36.0%)	9915 (28.0%)	9857 (27.9%)
NS_individual_15	36332	32857 (90.4%)	20494 (56.4%)	20463 (56.3%)	16475 (45.3%)	16428 (45.2%)
NS_individual_16	38665	34158 (88.3%)	19758 (51.1%)	19721 (51.0%)	14715 (38.1%)	14536 (37.6%)
NS_individual_17	32365	25542 (78.9%)	3492 (10.8%)	3490 (10.8%)	2473 (7.6%)	1669 (5.2%)
ND_pooled_1	145215	142835 (98.4%)	141970 (97.8%)	141692 (97.6%)	125588 (86.5%)	123239 (84.9%)
ND_pooled_2	117965	115997 (98.3%)	115655 (98.0%)	115462 (97.9%)	102679 (87.0%)	101164 (85.8%)
ND_pooled_3	84479	83182 (98.5%)	82949 (98.2%)	82794 (98.0%)	73153 (86.6%)	72050 (85.3%)
ND_pooled_4	74838	73605 (98.4%)	73217 (97.8%)	73077 (97.6%)	64903 (86.7%)	64094 (85.6%)
ND_pooled_5	42418	41667 (98.2%)	41447 (97.7%)	41381 (97.6%)	35361 (83.4%)	35066 (82.7%)
ND_pooled_6	36964	36224 (98.0%)	36030 (97.5%)	35955 (97.3%)	30483 (82.5%)	30004 (81.2%)
ND_pooled_7	43483	42725 (98.3%)	42545 (97.8%)	42476 (97.7%)	36056 (82.9%)	35604 (81.9%)
ND_pooled_8	31889	31403 (98.5%)	31235 (97.9%)	31175 (97.8%)	26354 (82.6%)	26080 (81.8%)
ND_pooled_9	104970	103444 (98.5%)	103253 (98.4%)	103101 (98.2%)	91371 (87.0%)	89782 (85.5%)
ND_pooled_10	99988	98511 (98.5%)	98500 (98.5%)	98362 (98.4%)	86828 (86.8%)	85140 (85.2%)
ND_pooled_11	86530	85342 (98.6%)	85331 (98.6%)	85210 (98.5%)	75421 (87.2%)	74060 (85.6%)
ND_pooled_12	85872	84691 (98.6%)	84677 (98.6%)	84550 (98.5%)	75081 (87.4%)	73684 (85.8%)
ND_pooled_13	53338	52595 (98.6%)	52381 (98.2%)	52296 (98.0%)	45670 (85.6%)	45170 (84.7%)
ND_pooled_14	53486	52690 (98.5%)	52514 (98.2%)	52420 (98.0%)	45643 (85.3%)	45068 (84.3%)

ND_pooled_15	35843	35301	35292	35241	30396	29654
		(98.5%)	(98.5%)	(98.3%)	(84.8%)	(82.7%)
SN_pooled_1	59780	58570	57426	57339	49113	48509
		(98.0%)	(96.1%)	(95.9%)	(82.2%)	(81.1%)
SN_pooled_2	50004	48709	47485	47403	38993	38070
		(97.4%)	(95.0%)	(94.8%)	(78.0%)	(76.1%)
SN_pooled_3	31041	30280	29325	29283	23791	23396
		(97.5%)	(94.5%)	(94.3%)	(76.6%)	(75.4%)
SN_pooled_4	36469	35524	34296	34255	27983	27359
		(97.4%)	(94.0%)	(93.9%)	(76.7%)	(75.0%)
SN_pooled_5	31194	30370	29240	29188	23612	22830
		(97.4%)	(93.7%)	(93.6%)	(75.7%)	(73.2%)
SN_pooled_6	32445	31454	30244	30188	24388	23956
		(96.9%)	(93.2%)	(93.0%)	(75.2%)	(73.8%)
SN_pooled_7	22492	21867	20893	20861	16743	16451
		(97.2%)	(92.9%)	(92.7%)	(74.4%)	(73.1%)
SN_pooled_8	16731	16284	15601	15579	12551	12345
		(97.3%)	(93.2%)	(93.1%)	(75.0%)	(73.8%)
SN_pooled_9	69650	67580	65346	65218	54691	53173
		(97.0%)	(93.8%)	(93.6%)	(78.5%)	(76.3%)
SN_pooled_10	69984	68305	67231	67084	55687	54419
		(97.6%)	(96.1%)	(95.9%)	(79.6%)	(77.8%)
SN_pooled_11	88670	86560	86529	86385	71377	68070
		(97.6%)	(97.6%)	(97.4%)	(80.5%)	(76.8%)
SN_pooled_12	78386	76530	76351	76202	63949	60622
		(97.6%)	(97.4%)	(97.2%)	(81.6%)	(77.3%)
SN_pooled_13	50454	49285	48675	48587	39035	38877
		(97.7%)	(96.5%)	(96.3%)	(77.4%)	(77.1%)
SN_pooled_14	40803	39743	39042	38966	30989	30218
		(97.4%)	(95.7%)	(95.5%)	(75.9%)	(74.1%)
SN_pooled_15	19301	18622	17872	17835	13482	13171
		(96.5%)	(92.6%)	(92.4%)	(69.9%)	(68.2%)
IB_pooled_1	63730	62832	62259	62163	49568	48406
		(98.6%)	(97.7%)	(97.5%)	(77.8%)	(76.0%)
IB_pooled_2	54408	53664	53246	53181	41821	40967
		(98.6%)	(97.9%)	(97.7%)	(76.9%)	(75.3%)
IB_pooled_3	45614	45044	44681	44628	35501	34683
		(98.8%)	(98.0%)	(97.8%)	(77.8%)	(76.0%)
IB_pooled_4	37609	37055	36624	36582	29616	28913
		(98.5%)	(97.4%)	(97.3%)	(78.7%)	(76.9%)

IB_pooled_5	35836	35330 (98.6%)	34923 (97.5%)	34865 (97.3%)	27523 (76.8%)	26710 (74.5%)
IB_pooled_6	37567	37053 (98.6%)	36632 (97.5%)	36588 (97.4%)	28582 (76.1%)	28019 (74.6%)
IB_pooled_7	29181	28739 (98.5%)	28320 (97.0%)	28283 (96.9%)	21068 (72.2%)	20678 (70.9%)
IB_pooled_8	15139	14915 (98.5%)	14798 (97.7%)	14771 (97.6%)	11095 (73.3%)	10897 (72.0%)
IB_pooled_9	81131	79198 (97.6%)	79052 (97.4%)	78944 (97.3%)	60401 (74.4%)	58259 (71.8%)
IB_pooled_10	44953	43855 (97.6%)	43776 (97.4%)	43700 (97.2%)	33444 (74.4%)	32261 (71.8%)
IB_pooled_11	46727	45519 (97.4%)	45275 (96.9%)	45201 (96.7%)	34290 (73.4%)	33030 (70.7%)
IB_pooled_12	44211	43139 (97.6%)	42606 (96.4%)	42529 (96.2%)	32651 (73.9%)	31916 (72.2%)
IB_pooled_13	45343	44248 (97.6%)	43768 (96.5%)	43708 (96.4%)	34469 (76.0%)	32358 (71.4%)
IB_pooled_14	48208	46973 (97.4%)	46408 (96.3%)	46317 (96.1%)	34247 (71.0%)	34618 (71.8%)
IB_pooled_15	14965	14382 (96.1%)	13504 (90.2%)	13479 (90.1%)	9233 (61.7%)	9033 (60.4%)
NS1_pooled_1	58009	56943 (98.2%)	56046 (96.6%)	55952 (96.5%)	47230 (81.4%)	46845 (80.8%)
NS1_pooled_2	76509	75021 (98.1%)	74397 (97.2%)	74282 (97.1%)	63339 (82.8%)	62427 (81.6%)
NS1_pooled_3	80656	79274 (98.3%)	78663 (97.5%)	78547 (97.4%)	66881 (82.9%)	66387 (82.3%)
NS1_pooled_4	57902	56885 (98.2%)	56293 (97.2%)	56203 (97.1%)	47742 (82.5%)	47440 (81.9%)
NS1_pooled_5	52588	51619 (98.2%)	50605 (96.2%)	50518 (96.1%)	42654 (81.1%)	42271 (80.4%)
NS1_pooled_6	56462	55444 (98.2%)	54728 (96.9%)	54624 (96.7%)	46122 (81.7%)	45731 (81.0%)
NS1_pooled_7	38588	37716 (97.7%)	36268 (94.0%)	36223 (93.9%)	29565 (76.6%)	29143 (75.5%)
NS1_pooled_8	14667	14360 (97.9%)	14009 (95.5%)	13982 (95.3%)	10997 (75.0%)	10929 (74.5%)
NS1_pooled_9	66187	65004 (98.2%)	64543 (97.5%)	64429 (97.3%)	53778 (81.3%)	53029 (80.1%)

NS1_pooled_10	65433	64287 (98.2%)	63935 (97.7%)	63835 (97.6%)	53314 (81.5%)	52843 (80.8%)
NS1_pooled_11	72041	70844 (98.3%)	70636 (98.0%)	70535 (97.9%)	59983 (83.3%)	59047 (82.0%)
NS1_pooled_12	54636	53692 (98.3%)	53133 (97.2%)	53044 (97.1%)	44161 (80.8%)	43903 (80.4%)
NS1_pooled_13	51397	50494 (98.2%)	50040 (97.4%)	49955 (97.2%)	42000 (81.7%)	41670 (81.1%)
NS1_pooled_14	53253	52216 (98.1%)	51428 (96.6%)	51320 (96.4%)	43010 (80.8%)	42569 (79.9%)
NS1_pooled_15	11781	11487 (97.5%)	11036 (93.7%)	11015 (93.5%)	8387 (71.2%)	8457 (71.8%)
NS2_pooled_1	10944	10721 (98.0%)	10360 (94.7%)	10349 (94.6%)	8143 (74.4%)	8156 (74.5%)
NS2_pooled_2	10898	10683 (98.0%)	10394 (95.4%)	10374 (95.2%)	8297 (76.1%)	8216 (75.4%)
NS2_pooled_3	10768	10561 (98.1%)	10233 (95.0%)	10224 (94.9%)	8150 (75.7%)	8167 (75.8%)
NS2_pooled_4	16696	16423 (98.4%)	16062 (96.2%)	16047 (96.1%)	13263 (79.4%)	13206 (79.1%)
NS2_pooled_5	27808	27312 (98.2%)	26467 (95.2%)	26422 (95.0%)	21962 (79.0%)	21920 (78.8%)
NS2_pooled_6	74708	73511 (98.4%)	72570 (97.1%)	72462 (97.0%)	62447 (83.6%)	61987 (83.0%)
NS2_pooled_7	58486	57109 (97.6%)	54612 (93.4%)	54537 (93.2%)	45856 (78.4%)	45329 (77.5%)
NS2_pooled_8	52815	51848 (98.2%)	50383 (95.4%)	50331 (95.3%)	42875 (81.2%)	42573 (80.6%)
NS2_pooled_9	10193	9922 (97.3%)	9711 (95.3%)	9691 (95.1%)	7453 (73.1%)	7434 (72.9%)
NS2_pooled_10	10052	9742 (96.9%)	9375 (93.3%)	9363 (93.1%)	7256 (72.2%)	7063 (70.3%)
NS2_pooled_11	10145	9825 (96.8%)	9420 (92.9%)	9407 (92.7%)	7387 (72.8%)	7160 (70.6%)
NS2_pooled_12	25839	25311 (98.0%)	25246 (97.7%)	25202 (97.5%)	20428 (79.1%)	20510 (79.4%)
NS2_pooled_13	30419	29559 (97.2%)	28575 (93.9%)	28521 (93.8%)	22757 (74.8%)	22569 (74.2%)
NS2_pooled_14	51848	50635 (97.7%)	49739 (95.9%)	49621 (95.7%)	40719 (78.5%)	40433 (78.0%)

NS2_pooled_15	141550	138517 (97.9%)	138204 (97.6%)	137995 (97.5%)	118736 (83.9%)	116856 (82.6%)
NS3_pooled_1	26361	25903 (98.3%)	25433 (96.5%)	25395 (96.3%)	20779 (78.8%)	20581 (78.1%)
NS3_pooled_2	14105	13793 (97.8%)	13436 (95.3%)	13423 (95.2%)	10422 (73.9%)	10298 (73.0%)
NS3_pooled_3	25413	24944 (98.2%)	24341 (95.8%)	24297 (95.6%)	19442 (76.5%)	19385 (76.3%)
NS3_pooled_4	48039	47222 (98.3%)	46625 (97.1%)	46541 (96.9%)	38670 (80.5%)	38351 (79.8%)
NS3_pooled_5	56711	55817 (98.4%)	55357 (97.6%)	55278 (97.5%)	46554 (82.1%)	46099 (81.3%)
NS3_pooled_6	96278	94615 (98.3%)	93888 (97.5%)	93706 (97.3%)	80837 (84.0%)	79960 (83.1%)
NS3_pooled_7	112154	110300 (98.3%)	108905 (97.1%)	108755 (97.0%)	94148 (83.9%)	92910 (82.8%)
NS3_pooled_8	1350	1319 (97.7%)	1222 (90.5%)	1221 (90.4%)	838 (62.1%)	823 (61.0%)
NS3_pooled_9	12210	11963 (98.0%)	11704 (95.9%)	11677 (95.6%)	9061 (74.2%)	8878 (72.7%)
NS3_pooled_10	15259	14913 (97.7%)	14595 (95.6%)	14570 (95.5%)	11293 (74.0%)	11312 (74.1%)
NS3_pooled_11	13988	13734 (98.2%)	13520 (96.7%)	13497 (96.5%)	10610 (75.9%)	10489 (75.0%)
NS3_pooled_12	50697	49818 (98.3%)	49414 (97.5%)	49340 (97.3%)	41140 (81.1%)	40808 (80.5%)
NS3_pooled_13	77706	76445 (98.4%)	76318 (98.2%)	76226 (98.1%)	64733 (83.3%)	63951 (82.3%)
NS3_pooled_14	98314	96691 (98.3%)	96269 (97.9%)	96134 (97.8%)	83091 (84.5%)	80997 (82.4%)
NS3_pooled_15	136528	134176 (98.3%)	134119 (98.2%)	133947 (98.1%)	114986 (84.2%)	114227 (83.7%)
ND_NegCon	41083	34540 (84.1%)	2538 (6.2%)	2532 (6.2%)	1768 (4.3%)	0 (0.0%)
SN_NegCon	49382	40223 (81.5%)	26 (0.1%)	25 (0.1%)	0 (0.0%)	0 (0.0%)
IB_NegCon	40144	33659 (83.8%)	11 (0.0%)	11 (0.0%)	0 (0.0%)	0 (0.0%)
NS_NegCon(1)	47655	39315 (82.5%)	112 (0.2%)	112 (0.2%)	60 (0.1%)	0 (0.0%)

NS_NegCon(2)	38881	32975 (84.8%)	10 (0.0%)	10 (0.0%)	0 (0.0%)	0 (0.0%)
NegCon.pcr(1)	67330	54264 (80.6%)	36 (0.1%)	34 (0.1%)	0 (0.0%)	0 (0.0%)
NegCon.pcr(2)	40300	34675 (86.0%)	3 (0.0%)	3 (0.0%)	0 (0.0%)	0 (0.0%)
total	9083566	8778402 (96.6%)	8152631 (89.8%)	8139675 (89.6%)	6801895 (84.8%)	6729952 (74.1%)

Table S2. List of fish species that are removed prior the community analyses.

Species	Total reads	Number of Samples detected	Annotation
Branchiostegus japonicus	12	1	Marine species
Lateolabrax japonicus	19	1	Marine species
Pagrus major	353	7	Marine species
Parapristipoma trilineatum	10	1	Marine species
Seriola dumerili	30	2	Marine species
Trachurus japonicus	15	1	Marine species
Saurogobio immaculatus	176	1	Not reported in Japan

Table S3. A data matrix depicting sequence reads of respective fish lineages in each sample. Sample numbers correspond to number of sampling positions and PCR replicates for the individual and pooled samples, respectively. For Nishinoko, three pooled samples are prepared, for one of which the filtration volume is doubled (pooled sample-3).

Fish lineages	Sample numbers																
	1	2	3	4	5	6	7	8	9	10	11	12	13	14	15	16	17
Nodanuma (individual sample)																	
Acheilognathus rhombeus	10	49	1455	16	0	457	269	10	24								
Anguilla japonica	0	0	0	16	0	0	0	0	0								
Biwia zezera	18	0	0	525	0	28	0	59	77								
Carassius cuvieri	672	1894	1157	2659	0	503	1177	238	1118								
Carassius spp.	6406	27256	6152	55000	217	32692	28324	23094	46637								
Channa argus	2999	3590	5623	809	71	14865	53759	92	132								
Cobitis biwae typeB	0	0	0	0	0	0	0	11	0								
Cottus reinii	10	0	0	0	0	0	0	23	0								
Cyprinus carpio	815	1105	1230	2459	18	10944	1379	1202	1374								
Gnathopogon caerulescens	125	1890	678	1372	18	10077	728	826	1331								
Gnathopogon elongatus elongatus	0	0	0	42	0	612	0	258	72								
Gymnogobius urotaenia	0	0	0	0	0	457	23	36	13								
Hemibarbus barbus	0	0	0	115	0	123	0	159	0								
Lepomis macrochirus	96754	28600	126804	9219	206902	35961	11432	1367	4196								
Micropterus spp.	13855	11219	6764	3346	1844	11698	7449	222	6485								
Nipponocypris sieboldii	17	0	34	116	0	2481	0	432	44								
Nipponocypris temminckii	0	0	0	0	0	0	0	26	51								
Odontobutis obscura	28	0	0	105	0	1153	0	641	53								
Opsariichthys uncirostris	0	17	43	128	0	1574	0	138	24								

Paramisgurnus / Misgurnus	0	20	0	0	0	564	33	105	0
Plecoglossus altivelis	0	0	0	0	0	0	0	21	0
Pseudogobio esocinus	0	0	0	115	0	140	0	214	53
Pseudorasbora parva	232	286	354	102	15	1455	275	0	83
Rhinogobius spp.	135	19	0	101	30	1808	0	357	68
Rhynchocypris lagowskii steindachneri	0	0	0	0	0	0	0	21	0
Sarcocheilichthys variegatus microoculus	0	0	0	22	0	1214	62	86	11
Silurus asotus	0	13	0	19	0	14	43	40	11
Tanakia lanceolata	0	0	0	0	0	0	0	20	0
Tanakia limbata	0	0	0	0	0	0	0	178	25
Tridentiger brevispinis	17	0	0	19	0	0	0	0	0
Zacco platypus	10	0	323	131	11	1593	0	265	616
Sonenuma (individual sample)									
Acheilognathus rhombeus	0	0	0	23	449	41	0	0	0
Biwia zezera	0	0	0	3409	777	134	46	0	0
Carassius cuvieri	4987	40	4674	483	112	43	0	23	368
Carassius spp.	36116	634	34240	10194	4792	1720	1158	1444	15798
Channa argus	17372	2704	3381	686	3568	271	1747	512	122
Cyprinus carpio	1405	1360	3111	4304	6025	1671	2305	1464	709
Gnathopogon caerulescens	1044	167	0	8839	16	123899	53	935	68
Gymnogobius isaza	0	0	15	0	0	0	0	0	0
Gymnogobius urotaenia	2538	4013	277	5630	7239	321	4034	2617	746
Hemibarbus barbus	0	0	0	23	0	0	0	0	0
Lepomis macrochirus	47430	58642	39976	43231	23580	5124	145408	24002	8438
Micropterus spp.	893	0	154	881	623	99	0	0	0

Nipponocypris sieboldii	0	0	0	25	0	0	0	0	0
Odontobutis obscura	0	0	0	638	0	0	0	0	0
Opsariichthys uncirostris	0	0	0	234	528	52	0	0	0
Paramisgurnus / Misgurnus	0	271	0	36	50	84	263	0	0
Plecoglossus altivelis	0	0	0	17	0	0	0	0	0
Pseudorasbora parva	2235	1518	201	1264	585	189	1563	11272	0
Rhinogobius spp.	96	0	0	481	4196	205	232	3528	12
Silurus asotus	0	0	0	24	0	62	0	0	15
Tridentiger brevispinis	0	0	0	0	0	43	0	47	0
Zacco platypus	381	193	0	5369	79	19	195	1403	0
**Ibanaiko (individual sample)**									
Acheilognathus rhombeus	36	1150	26	12083	0	188	0	54	0
Biwia zezera	670	965	44	0	38879	5835	10872	1763	3943
Carassius cuvieri	3304	27	42	3826	0	1034	391	94	38857
Carassius spp.	29901	28964	7918	18632	4008	12308	6072	4883	10884
Channa argus	369	2395	400	4405	0	5861	105	726	0
Cobitis biwae typeB	0	0	0	0	0	0	18	0	0
Cottus reinii	0	0	0	0	1048	0	0	0	0
Cyprinus carpio	1697	1095	406	13697	336	1017	2148	4525	1473
Gnathopogon caerulescens	1040	11266	1716	109	1388	5573	7545	14626	5834
Gnathopogon elongatus elongatus	0	0	0	0	1136	0	0	0	0
Gymnogobius urotaenia	0	531	14	187	106	0	244	27	0
Hemibarbus barbus	0	0	0	57	3836	508	415	0	0
Ischikauia steenackeri	0	0	0	0	0	0	0	0	808
Lepomis macrochirus	53558	2624	84632	34904	13	12759	11397	30240	1925

Micropterus spp.	3892	1622	8544	20261	0	6671	2469	10600	0								
Nipponocypris sieboldii	0	100	20	0	211	0	0	0	0								
Nipponocypris temminckii	0	0	0	0	5260	0	390	0	0								
Odontobutis obscura	39	122	0	1186	781	0	109	78	0								
Opsariichthys uncirostris	16	1003	41	0	177	26	0	94	2155								
Paramisgurnus / Misgurnus	0	108	26	841	74	13	50	10	0								
Pseudogobio esocinus	44	0	0	14	6850	277	4078	0	0								
Pseudorasbora parva	0	0	0	0	103	0	40	0	0								
Pungtungia herzi	0	0	0	0	4992	22	459	0	0								
Rhinogobius spp.	257	3533	143	14	1989	520	773	587	55								
Rhodeus ocellatus ocellatus	0	0	10	0	0	0	0	0	0								
Rhynchocypris lagowskii steindachneri	23	140	0	116	6080	0	1246	0	0								
Sarcocheilichthys variegatus microoculus	314	367	0	0	0	222	529	162	0								
Sarcocheilichthys variegatus variegatus	0	11	0	0	0	21	0	0	0								
Silurus asotus	0	12	14	29	0	0	0	17	0								
Squalidus chankaensis	0	0	0	0	3548	305	479	36	0								
Tanakia limbata	0	64	0	0	25	0	19	0	0								
Tridentiger brevispinis	171	430	23	0	38	289	82	9074	0								
Zacco platypus	53	44	92	0	918	535	1016	0	0								
**Nishinoko (individual)**																	
Acheilognathus rhombeus	0	0	0	0	49	0	0	0	0	25	1490	201	0	33	0	0	0
Biwia zezera	4109	85	2656	1246	0	745	167	795	119525	34438	595	6964	12110	103	206	3896	0
Carassius cuvieri	3422	1165	35	394	306	0	210	281	24	552	24	20	578	30	46	160	0
Carassius spp.	1654	3434	3239	2436	10979	303	246	737	2787	8242	2730	349	3529	563	643	1330	71
Channa argus	15	98	107	125	20	82	0	61	33	0	0	0	77	41	0	26	0

Ctenopharyngodon idella	0	57	0	0	0	0	0	0	0	0	0	0	0	0	0	0	0
Cyprinus carpio	927	822	4637	786	329	303	45	527	436	9186	11442	131	4110	2735	76	745	338
Gnathopogon caerulescens	433	600	8600	1557	434	38	0	202	9431	3707	2280	5595	2080	78	817	1565	730
Gnathopogon elongatus elongatus	0	0	0	0	0	0	0	32	0	0	0	0	0	0	0	0	0
Hemibarbus barbus	2019	102	443	0	50	0	119	150	347	845	321	29	5148	50	0	1346	0
Hypomesus nipponensis	0	0	0	0	0	0	0	0	0	72	0	0	0	0	0	0	0
Hypophthalmichthys molitrix	0	0	0	0	0	0	0	0	0	0	0	0	168	0	0	0	0
Hypophthalmichthys nobilis	696	0	0	56	0	0	0	0	0	0	0	0	736	0	0	137	72
Lepomis macrochirus	17222	3578	5857	14083	153365	9982	16830	6624	1956	1966	10231	6955	8458	4837	11353	3356	0
Micropterus spp.	1220	207	188	773	1988	284	666	233	124	1158	428	284	1133	387	1965	105	61
Nipponocypris sieboldii	0	0	0	0	0	0	175	0	230	0	0	0	153	0	0	0	0
Odontobutis obscura	123	0	0	0	0	0	0	0	0	0	0	0	116	0	0	19	0
Opsariichthys uncirostris	457	0	0	0	125	263	0	0	27	249	136	0	399	0	0	317	238
Paramisgurnus / Misgurnus	0	0	16	0	0	0	0	20	0	0	37	0	63	0	0	0	0
Pseudogobio esocinus	163	0	0	0	0	43	0	0	31	60	0	206	630	0	0	101	0
Pseudorasbora parva	101	0	236	87	308	177	44	147	0	0	65	54	0	251	934	107	62
Pungtungia herzi	0	0	0	0	0	0	0	0	0	0	0	0	71	0	0	0	0
Rhinogobius spp.	497	267	785	398	61	38	28	0	18	77	553	381	629	10	116	300	0
Rhodeus ocellatus ocellatus	148	0	0	93	0	0	25	0	0	0	0	0	0	0	0	0	0
Sarcocheilichthys variegatus microoculus	406	71	132	18	0	0	0	32	23	0	96	135	0	0	0	90	0
Sarcocheilichthys variegatus variegatus	0	0	0	0	0	0	0	80	0	0	0	0	0	0	0	0	0
Silurus asotus	0	0	0	0	0	0	0	0	0	0	18	0	26	0	0	0	0
Squalidus chankaensis	453	0	0	74	0	0	58	40	119	64	231	206	1266	465	28	476	0
Tanakia limbata	0	0	0	0	0	0	0	0	0	0	0	0	23	0	0	0	0
Tridentiger brevispinis	0	80	813	176	0	58	42	0	453	0	121	24	44	0	244	79	0

Zacco platypus	0	0	0	349	102	429	312	1918	363	1127	287	383	1052	274	0	381	97
<u>Nodanuma (pooled sample)</u>																	
Acheilognathus rhombeus	80	47	28	41	23	47	19	26	79	234	101	48	66	34	63		
Biwia zezera	68	109	99	93	171	179	58	91	85	19	44	44	89	0	0		
Carassius cuvieri	912	438	141	444	120	164	94	67	261	433	125	367	49	123	228		
Carassius spp.	10808	8448	3951	4149	2355	2412	3028	1705	8328	8319	6894	6682	3305	3659	3127		
Channa argus	444	437	709	535	292	222	398	108	1379	1785	771	1403	266	283	548		
Cottus reinii	0	0	0	0	0	0	0	0	0	17	0	0	0	0	0		
Cyprinus carpio	357	1161	290	178	135	103	172	171	855	469	744	511	290	185	186		
Gnathopogon caerulescens	271	431	351	111	61	93	90	101	304	409	367	443	112	205	82		
Gnathopogon elongatus elongatus	0	0	18	0	57	0	22	0	25	102	0	63	11	0	0		
Gymnogobius urotaenia	0	19	10	0	0	0	0	0	0	44	0	56	0	48	36		
Hemibarbus barbus	0	0	0	0	0	18	0	10	20	0	0	0	0	0	0		
Lepomis macrochirus	105751	87901	63202	57293	30913	25854	31086	23058	75943	68916	60736	61294	39717	39023	24114		
Micropterus spp.	3532	1704	2884	1037	772	737	480	614	1991	3583	3364	2449	983	1159	933		
Nipponocypris sieboldii	0	39	0	0	0	0	0	0	0	0	27	64	0	11	12		
Nipponocypris temminckii	90	0	0	0	0	0	0	0	34	0	0	0	0	0	0		
Odontobutis obscura	40	26	46	45	0	38	12	22	43	44	176	75	17	75	10		
Opsariichthys uncirostris	0	0	71	82	12	0	20	0	0	125	133	55	0	0	44		
Paramisgurnus / Misgurnus	85	0	31	0	0	0	0	18	0	24	41	0	0	0	40		
Plecoglossus altivelis	0	0	0	0	0	0	0	0	0	0	14	0	0	0	0		
Pseudogobio esocinus	152	47	49	15	32	21	0	0	0	101	0	42	24	21	0		
Pseudorasbora parva	106	152	104	40	21	51	51	0	163	113	80	0	58	102	23		
Rhinogobius spp.	375	151	53	13	0	0	74	0	155	179	194	39	98	57	74		
Rhynchocypris lagowskii steindachneri	0	0	0	0	0	0	0	0	0	41	131	0	0	0	0		

Sarcocheilichthys variegatus microoculus	0	0	0	0	0	0	0	0	0	26	0	0	0	0	0
Silurus asotus	31	0	0	0	0	0	0	0	0	0	12	0	0	0	0
Squalidus chankaensis	0	0	0	0	0	0	0	0	0	0	0	0	22	0	0
Tanakia lanceolata	69	11	0	0	0	0	0	0	0	34	0	0	0	13	31
Tanakia limbata	68	43	13	18	13	17	0	22	36	26	17	37	51	70	10
Tridentiger brevispinis	0	0	0	0	10	15	0	0	0	0	0	0	0	0	0
Zacco platypus	0	0	0	0	79	33	0	67	81	97	89	12	12	0	93
**Sonenuma (pooled sample)**															
Acheilognathus rhombeus	0	0	0	0	0	0	0	0	0	13	108	18	48	0	22
Biwia zezera	0	442	10	12	0	87	149	96	418	414	1292	524	475	204	285
Carassius cuvieri	404	190	266	544	0	62	58	201	512	481	659	352	93	119	374
Carassius spp.	4650	5715	2346	3435	3296	3803	1347	1239	4644	4907	8676	9938	3920	4211	1139
Channa argus	4845	3670	1813	1750	1636	2013	630	615	3202	3881	8373	6912	3495	2362	633
Cyprinus carpio	290	438	800	1048	860	1267	573	640	1255	2119	3288	2579	1404	609	503
Gnathopogon caerulescens	264	757	302	334	368	329	39	119	321	326	1016	1334	415	586	31
Gymnogobius urotaenia	212	321	255	372	308	173	144	142	325	316	3326	982	265	756	262
Lepomis macrochirus	37272	25494	17283	19181	15516	15828	13169	9128	42223	41831	38354	36327	28217	20818	9616
Micropterus spp.	0	87	0	59	76	0	58	0	0	0	0	199	169	0	40
Nipponocypris sieboldii	0	0	0	0	0	0	20	0	0	0	0	0	0	0	0
Odontobutis obscura	0	0	0	63	0	0	31	0	0	0	0	0	0	33	20
Opsariichthys uncirostris	0	220	0	0	124	62	0	0	45	0	0	60	0	0	0
Paramisgurnus / Misgurnus	145	0	0	54	0	101	0	33	62	26	514	423	155	115	47
Pseudorasbora parva	40	428	144	278	443	205	134	71	84	65	1139	652	189	212	131
Rhinogobius flumineus	0	0	0	12	0	0	0	0	0	0	0	0	0	0	0
Rhinogobius spp.	256	0	127	0	115	26	72	26	0	40	475	258	32	85	0

Silurus asotus	0	0	0	0	10	0	0	0	0	0	0	0	0	0	0
Tridentiger brevispinis	0	0	0	0	0	0	0	0	0	0	0	25	0	0	0
Zacco platypus	131	308	50	217	78	0	27	35	82	0	850	39	0	108	68
<u>Ibanaiko (pooled sample)</u>															
Acheilognathus rhombeus	746	519	551	179	291	216	308	346	1127	533	586	626	527	767	148
Anguilla japonica	0	0	0	0	0	0	0	0	34	0	0	0	0	0	0
Biwia zezera	5551	5136	3007	2549	2546	3430	1730	1392	7152	3671	3606	3522	2481	3669	1192
Carassius cuvieri	632	242	612	47	457	257	142	123	935	197	682	173	225	838	91
Carassius spp.	7850	7197	6184	5153	5374	4325	3785	1787	10682	5592	6017	5569	5447	5545	1518
Channa argus	849	275	164	478	268	749	227	263	543	1490	469	487	449	756	139
Cobitis biwae typeB	48	0	0	0	0	0	0	0	0	0	0	0	0	0	0
Cottus reinii	161	246	23	51	32	123	68	39	165	109	39	114	107	219	51
Cyprinus carpio	927	749	505	1209	847	1188	437	365	2589	1160	1333	570	1037	1084	169
Gnathopogon caerulescens	3749	3219	3274	1360	2128	2283	2544	814	4942	2679	3060	2611	2438	2410	446
Gnathopogon elongatus elongatus	123	109	157	45	240	15	28	82	858	131	380	0	64	38	26
Gymnogobius urotaenia	46	0	0	56	0	97	15	14	91	0	15	35	43	81	0
Hemibarbus barbus	925	774	1110	328	342	685	580	116	871	975	461	534	519	376	96
Lepomis macrochirus	17939	13037	12595	11196	9038	9227	6699	3491	13899	7652	9356	11353	11773	12898	3847
Micropterus spp.	3162	3710	2582	3368	2562	2042	1767	795	6560	4282	3228	2460	2843	1945	615
Nipponocypris sieboldii	0	0	0	0	0	18	0	52	0	0	0	0	72	0	0
Nipponocypris temminckii	334	104	66	178	0	229	88	78	486	127	251	59	174	320	31
Odontobutis obscura	339	315	79	17	98	66	190	73	391	169	294	191	65	120	12
Opsariichthys uncirostris	0	17	0	0	0	0	0	29	0	131	0	0	74	22	0
Paramisgurnus / Misgurnus	185	72	83	227	140	136	152	35	107	152	118	73	289	69	26
Pseudogobio esocinus	446	753	424	166	129	203	36	57	562	595	579	106	177	158	52

Pungtungia herzi	1260	1012	661	329	284	453	262	103	421	430	210	651	1131	505	54
Rhinogobius spp.	858	1727	984	647	750	1128	684	309	1961	1021	760	1644	752	1266	275
Rhynchocypris lagowskii steindachneri	1316	1036	724	593	727	519	315	304	2185	698	793	542	822	589	141
Sarcocheilichthys variegatus microoculus	42	0	201	213	0	0	102	17	149	57	0	0	71	0	0
Squalidus chankaensis	250	126	181	26	108	82	190	22	357	15	73	39	201	0	32
Tanakia limbata	0	0	25	51	90	0	0	0	0	38	127	97	85	26	0
Tridentiger brevispinis	480	466	299	371	169	475	308	62	726	327	593	180	291	470	33
Zacco platypus	188	126	192	76	90	54	21	129	466	30	0	280	158	447	39
<u>**Nishinoko (pooled sample-1)**</u>															
Acheilognathus rhombeus	90	65	45	55	83	25	12	13	0	51	130	190	70	0	0
Biwia zezera	31382	44854	41340	32708	25592	30658	19329	7699	35610	35049	38283	26410	28751	27687	5320
Carassius cuvieri	149	0	74	0	0	0	40	22	157	110	29	11	92	144	0
Carassius spp.	1112	1191	2178	1643	1279	1787	523	245	2421	1379	2160	627	1103	1359	217
Channa argus	75	220	232	65	74	0	31	13	26	0	18	40	0	55	11
Ctenopharyngodon idella	62	0	0	0	0	0	0	0	0	0	0	0	0	0	0
Cyprinus carpio	649	1575	955	456	899	771	545	96	222	1029	1401	1283	866	310	57
Gnathopogon caerulescens	2295	3260	3953	1282	1142	1762	968	428	1702	3331	2600	2191	2292	1350	232
Hemibarbus barbus	135	646	446	202	92	100	106	60	393	296	295	123	45	62	29
Hypomesus nipponensis	0	0	0	0	0	0	0	0	10	0	0	0	0	0	0
Hypophthalmichthys nobilis	0	100	0	115	0	0	30	0	0	137	0	0	0	0	0
Lepomis macrochirus	9744	8275	15812	9002	11932	9228	6409	2134	10689	9592	11838	11149	7158	10622	2406
Micropterus spp.	227	807	467	382	338	388	182	0	867	369	432	356	352	67	37
Nipponocypris sieboldii	0	0	0	140	0	0	0	0	115	0	98	50	28	0	0
Odontobutis obscura	0	0	0	0	0	0	0	0	0	0	33	0	0	0	0
Opsariichthys uncirostris	23	446	259	264	0	0	132	10	56	54	281	77	0	49	33

Paramisgurnus / Misgurnus	0	0	0	38	0	16	20	0	25	12	50	0	0	0	0
Pseudogobio esocinus	203	0	0	0	0	0	54	0	172	129	0	29	17	70	0
Pseudorasbora parva	285	75	307	298	165	345	114	83	120	130	454	431	279	324	13
Pungtungia herzi	0	0	0	0	0	15	48	0	0	0	0	0	0	0	0
Rhinogobius spp.	18	184	82	0	97	31	145	29	82	158	77	31	26	47	22
Rhodeus ocellatus ocellatus	69	0	92	0	0	60	0	0	71	58	0	0	0	16	0
Sarcocheilichthys variegatus microoculus	0	0	0	0	10	142	60	12	77	0	146	224	0	19	0
Squalidus chankaensis	205	95	80	170	29	16	72	59	123	225	0	192	59	0	12
Tribolodon hakonensis	0	0	0	0	27	0	0	0	0	0	0	0	0	0	0
Tridentiger brevispinis	0	72	0	138	141	163	86	26	76	139	386	213	183	253	36
Zacco platypus	122	562	65	482	371	224	237	0	15	595	336	257	349	135	32
Nishinoko (pooled sample-2)															
Acheilognathus rhombeus	0	0	0	0	23	0	0	0	0	0	12	21	13	21	53
Anguilla japonica	0	0	0	0	21	0	0	0	0	0	0	0	0	0	0
Biwia zezera	3781	3718	4096	7019	12720	42114	26559	25375	2787	3210	3274	10750	11180	24556	77074
Carassius cuvieri	21	24	0	29	74	33	121	193	33	17	0	64	162	153	16
Carassius spp.	286	352	343	383	512	1404	2342	1207	259	331	342	854	736	1357	4924
Channa argus	29	43	18	36	15	16	408	0	45	72	41	168	83	242	654
Cyprinus carpio	80	91	84	75	300	559	1138	843	56	91	66	258	259	328	1706
Gnathopogon caerulescens	210	178	198	368	906	2593	1388	2119	206	210	143	729	690	2394	3641
Gymnogobius urotaenia	0	0	0	0	0	0	0	0	0	0	0	11	0	0	0
Hemibarbus barbus	52	13	59	58	79	259	0	113	0	0	0	100	177	77	1232
Hypomesus nipponensis	13	0	0	0	0	0	0	0	0	0	0	0	0	0	0
Hypophthalmichthys nobilis	0	0	0	0	0	0	0	0	0	0	0	0	11	0	0
Ischikauia steenackeri	0	0	0	0	0	0	51	0	0	0	0	0	0	0	0

Lepomis macrochirus	3503	3577	3157	5029	6879	13953	11553	11997	3917	2953	3090	6485	8722	10089	25024
Micropterus spp.	55	111	128	35	116	392	799	225	73	115	79	212	336	238	632
Nipponocypris sieboldii	0	0	0	0	0	0	0	0	0	0	0	0	0	0	69
Odontobutis obscura	0	0	0	0	0	0	0	0	0	0	0	0	22	11	0
Opsariichthys uncirostris	11	11	0	0	0	85	0	26	0	0	18	40	0	99	208
Paramisgurnus / Misgurnus	0	0	0	0	12	0	53	68	0	0	0	16	0	0	0
Pseudogobio esocinus	0	0	0	0	0	25	0	0	0	0	0	49	14	0	39
Pseudorasbora parva	28	22	41	32	62	154	379	142	0	32	34	107	66	136	840
Rhinogobius spp.	48	18	0	10	15	171	125	150	15	10	17	135	74	121	215
Rhodeus ocellatus ocellatus	12	0	0	0	0	0	0	56	0	0	0	0	0	0	61
Sarcocheilichthys variegatus microoculus	0	0	0	0	0	0	0	0	0	0	15	0	0	0	0
Squalidus chankaensis	15	0	14	28	17	97	35	0	0	22	19	64	0	0	113
Tridentiger brevispinis	0	23	29	21	90	42	62	0	27	0	0	70	11	183	53
Zacco platypus	12	35	0	83	79	90	316	59	16	0	10	184	13	428	302
Nishinoko (pooled sample-3)															
Acheilognathus rhombeus	32	32	0	0	37	65	55	0	0	15	16	29	195	0	359
Biwia zezera	9956	5037	9363	20830	21554	45517	50022	30	4288	6168	5591	24704	36146	45313	64480
Carassius cuvieri	50	15	65	122	100	386	200	10	28	0	40	62	19	411	368
Carassius spp.	588	475	770	1122	1079	3552	3827	55	286	356	477	1147	2175	2742	5315
Channa argus	171	100	100	198	114	126	403	32	61	48	34	53	79	31	593
Cyprinus carpio	293	186	271	468	1024	1835	1983	24	123	78	147	652	1163	2189	4858
Gnathopogon caerulescens	518	341	649	1958	1754	2492	3511	14	295	526	418	1819	3330	2604	5659
Gymnogobius urotaenia	11	15	16	34	31	12	15	13	0	0	12	10	0	0	0
Hemibarbus barbus	119	55	83	275	259	359	269	0	19	49	81	164	345	455	877
Hypophthalmichthys molitrix	0	0	0	0	0	0	0	0	0	0	0	0	19	0	0

Hypophthalmichthys nobilis	0	0	11	23	0	0	0	0	0	0	0	0	28	0	0
Ischikauia steenackeri	0	0	0	0	0	0	0	0	11	0	0	0	0	0	0
Lepomis macrochirus	8249	3690	7392	11987	18336	22435	30108	645	3411	3684	3456	11092	18099	24089	25969
Micropterus spp.	249	109	164	352	378	1547	1514	0	135	121	70	255	665	1041	1331
Nipponocypris sieboldii	11	0	50	0	21	0	68	0	0	16	0	0	0	39	14
Odontobutis obscura	0	0	0	0	0	28	0	0	10	0	0	0	0	0	0
Opsariichthys uncirostris	64	15	0	0	133	50	0	0	19	17	0	0	95	11	171
Paramisgurnus / Misgurnus	0	0	0	0	0	106	0	0	0	0	0	0	39	51	0
Pseudogobio esocinus	0	10	0	0	26	45	0	0	0	0	0	0	82	0	20
Pseudorasbora parva	79	58	112	433	260	184	110	0	51	96	48	314	456	384	941
Rhinogobius spp.	59	0	126	140	222	185	28	0	30	38	23	38	223	147	860
Rhodeus ocellatus ocellatus	0	12	27	0	26	0	0	0	0	18	0	0	0	36	0
Sarcocheilichthys variegatus microoculus	16	0	0	23	0	65	0	0	0	0	0	0	0	35	0
Silurus asotus	0	0	0	0	0	21	0	0	0	0	0	0	0	0	0
Squalidus chankaensis	22	0	39	53	103	97	79	0	34	0	15	129	64	275	866
Tridentiger brevispinis	50	28	23	140	270	439	171	0	56	34	23	224	435	361	397
Zacco platypus	44	120	103	193	372	414	547	0	21	48	38	116	294	726	995

Table S4. Geographical locations and environmental conditions of respective sampling locations.

Sample id	Geographical location	pH	Electrical conductivity	Temperate
Nodanuma				
ND_individual_1	35.2475°N, 136.2080°E	7.19	0.23	21.4
ND_individual_2	35.2478°N, 136.2066°E	7.11	0.23	21.1
ND_individual_3	35.2493°N, 136.2063°E	7.17	0.21	21.2
ND_individual_4	35.2487°N, 136.2076°E	7.29	0.23	21
ND_individual_5	35.2489°N, 136.2080°E	7.25	0.23	20.8
ND_individual_6	35.2501°N, 136.2073°E	6.91	0.25	21.2
ND_individual_7	35.2499°N, 136.2086°E	6.95	0.23	20.7
ND_individual_8	35.2491°N, 136.2095°E	7.3	0.22	20
ND_individual_9	35.2483°N, 136.2070°E	7.09	0.24	21.4
Sonenuma				
SN_individual_1	35.2429°N, 136.1989°E	7.16	0.27	21.8
SN_individual_2	35.2414°N, 136.1967°E	6.88	0.23	20.5
SN_individual_3	35.2390°N, 136.1951°E	7.05	0.19	22.5
SN_individual_4	35.2392°N, 136.1928°E	7.36	0.22	21.9
SN_individual_5	35.2412°N, 136.1903°E	7.23	0.22	22.5
SN_individual_6	35.2429°N, 136.1923°E	7.34	0.21	22.6
SN_individual_7	35.2424°N, 136.1946°E	7.4	0.23	22.2
SN_individual_8	35.2431°N, 136.1965°E	7.54	0.26	22.3
SN_individual_9	35.2409°N, 136.1941°E	7.11	0.24	22.9
Ibanaiko				
IB_individual_1	35.1879°N, 136.1355°E	7.56	0.14	22.4
IB_individual_2	35.1897°N, 136.1398°E	7.57	0.17	22.4

IB_individual_3	35.1873°N, 136.1422°E	7.86	0.14	23
IB_individual_4	35.1846°N, 136.1453°E	6.82	0.15	23.4
IB_individual_5	35.1820°N, 136.1461°E	7.24	0.14	19.5
IB_individual_6	35.1808°N, 136.1409°E	7.59	0.15	23.2
IB_individual_7	35.1763°N, 136.1378°E	7.44	0.13	22.5
IB_individual_8	35.1838°N, 136.1388°E	8.02	0.14	23.8
IB_individual_9	35.1847°N, 136.1413°E	9.16	0.13	22.3
Nishinoko				
NS_individual_1	35.1635°N, 136.1041°E	7.27	0.2	24.4
NS_individual_2	35.1623°N, 136.1091°E	7.24	0.2	25.1
NS_individual_3	35.1638°N, 136.1134°E	7.18	0.2	24.4
NS_individual_4	35.1627°N, 136.1185°E	7.29	0.2	26.3
NS_individual_5	35.1649°N, 136.1216°E	7.14	0.19	26.4
NS_individual_6	35.1602°N, 136.1231°E	7.04	0.2	27
NS_individual_7	35.1570°N, 136.1262°E	6.87	0.19	27.4
NS_individual_8	35.1533°N, 136.1292°E	7.04	0.19	27
NS_individual_9	35.1521°N, 136.1246°E	7.87	0.19	26.2
NS_individual_10	35.1541°N, 136.1198°E	8.07	0.19	25.8
NS_individual_11	35.1525°N, 136.1141°E	8.1	0.2	26.3
NS_individual_12	35.1553°N, 136.1098°E	8.09	0.2	26.2
NS_individual_13	35.1585°N, 136.1045°E	8.34	0.19	25.5
NS_individual_14	35.1554°N, 136.0988°E	6.8	0.18	26.2
NS_individual_15	35.1586°N, 136.1008°E	7.14	0.18	24.9
NS_individual_16	35.1636°N, 136.0994°E	7.46	0.19	24.5
NS_individual_17	35.1582°N, 136.1142°E	8.05	0.19	25.4

Table S5. The united lineage name for species that shared identical sequences.

United lineage name	Original species name in the reference database	Note
Carassius cuvieri	Carassius auratus x Megalobrama amblycephala pentaploid hybrid	*2
Carassius spp.	Carassius auratus auratus	*1
	Carassius auratus var WAKIN	*1
	Carassius auratus langsdorfii	*1
	Carassius auratus grandoculis	*1
	Carassius sp HUMZ 222861	*1
	Carassius sp CBM ZF 15597 1	*1
	Carassius auratus subsp KINBUNA	*1
	Carassius gibelio	*1
	Carassius auratus buergeri	*1
	Carassius sp CBM ZF 11717 Tonegawa River	*1
	Carassius auratus subsp Okinawa Is	*1
	Carassius auratus subsp Ishigaki Is	*1
	Carassius auratus	*1
	Carassius auratus ssp. 'Pingxiang'	*1
	Carassius auratus x Cyprinus carpio	*1
	Carassius carassius	*1
	Carassius auratus x Cyprinus carpio x Carassius cuvieri	*1
	Carassius auratus x Megalobrama amblycephala tetraploid hybrid	*1
	Carassius auratus x Megalobrama amblycephala triploid hybrid	*1
	Carassius auratus 'high back crucian carp'	*1
	natural gynogenetic Carassius auratus red var.	*1
Ctenopharyngodon idella	Ctenopharyngodon idella x Squaliobarbus curriculus	*1
	Ctenopharyngodon idella x Megalobrama amblycephala	*1

	Ctenopharyngodon idellus x Elopichthys bambusa	*1
Cyprinus carpio	Cyprinus carpio 'Furong' x Carassius auratus red var.	*1
	Cyprinus carpio specularis x Carassius cuvieri	*1
	Cyprinus carpio 'koi'	*1
	Cyprinus carpio carpio	*1
	Cyprinus carpio 'xingguonensis'	*1
	Cyprinus carpio haematopterus	*1
	Cyprinus carpio wuyuanensis x Carassius auratus	*1
	Cyprinus carpio 'wuyuanensis'	*1
	Cyprinus megalophthalmus	*1
	Cyprinus carpio 'wananensis'	*1
	Cyprinus carpio 'longfin'	*1
	Cyprinus carpio 'color'	*1
	Cyprinus carpio nudus	*1
	Procypris mera	*2
Gymnogobius urotaenia	Gymnogobius opperiens	*2
Hemibarbus barbus	Hemibarbus medius	*2
	Hemibarbus umbrifer	*2
Hypomesus nipponensis	Hypomesus olidus	*2
Micropterus spp.	Micropterus floridanus	*1
	Micropterus salmoides	*1
Paramisgurnus/Misgurnus	Misgurnus anguillicaudatus	*1
	Paramisgurnus dabryanus ssp. DLY-2014	*1
	Misgurnus mizolepis	*1
	Pseudogobio esocinus Clade A	*1
	Pseudogobio esocinus esocinus	*1

Rhinogobius spp.	Rhinogobius brunneus	*1
	Rhinogobius sp CBM ZF 12364	*1
	Rhinogobius sp OGASAWARA YOSHINOBORI	*1
	Rhinogobius sp CBM ZF 12303	*1
	Rhinogobius sp CBM ZF 13662	*1
	Rhinogobius sp CBM ZF 13663	*1
	Rhinogobius sp CBM ZF 14538	*1
	Rhinogobius gigas	*1
	Rhinogobius sp CBM ZF 12365	*1
	Rhinogobius sp OR	*1
	Rhinogobius kurodai	*1
	Rhinogobius sp OR Kyoto	*1
	Rhinogobius sp BW	*1
	Rhinogobius sp OM	*1
	Rhinogobius sp OR Nagano	*1
	Rhinogobius nagoyae	*1
	Rhinogobius sp CO	*1
	Rhinogobius fluviatilis	*1
	Rhinogobius sp. HYW-2001	*1
Squalidus chankaensis	Squalidus chankaensis biwae	*1
	Squalidus chankaensis tsuchigae	*1
Tridentiger brevispinis	Tridentiger obscurus	*1
	Tridentiger kuroiwae	*1

*1: Closely related species that share identical sequences were merged

*2: Species that are unlikely to inhabit the study areas were removed
